# Host restriction of emerging high-pathogenic bunyaviruses via MOV10 by targeting viral nucleoprotein and blocking ribonucleoprotein assembly

**DOI:** 10.1371/journal.ppat.1009129

**Published:** 2020-12-07

**Authors:** Qiong Mo, Zhao Xu, Fei Deng, Hualin Wang, Yun-Jia Ning

**Affiliations:** 1 State Key Laboratory of Virology, Wuhan Institute of Virology, Chinese Academy of Sciences, Wuhan, China; 2 University of Chinese Academy of Sciences, Beijing, China; 3 Center for Biosafety Mega-Science, Chinese Academy of Sciences, Wuhan, China; MRC University of Glasgow Centre for Virus Research, UNITED KINGDOM

## Abstract

Bunyavirus ribonucleoprotein (RNP) that is assembled by polymerized nucleoproteins (N) coating a viral RNA and associating with a viral polymerase can be both the RNA synthesis machinery and the structural core of virions. Bunyaviral N and RNP thus could be assailable targets for host antiviral defense; however, it remains unclear which and how host factors target N/RNP to restrict bunyaviral infection. By mass spectrometry and protein-interaction analyses, we here show that host protein MOV10 targets the N proteins encoded by a group of emerging high-pathogenic representatives of bunyaviruses including severe fever with thrombocytopenia syndrome virus (SFTSV), one of the most dangerous pathogens listed by World Health Organization, in RNA-independent manner. MOV10 that was further shown to be induced specifically by SFTSV and related bunyaviruses in turn inhibits the bunyaviral replication in infected cells in series of loss/gain-of-function assays. Moreover, animal infection experiments with MOV10 knockdown corroborated the role of MOV10 in restricting SFTSV infection and pathogenicity *in vivo*. Minigenome assays and additional functional and mechanistic investigations demonstrate that the anti-bunyavirus activity of MOV10 is likely achieved by direct impact on viral RNP machinery but independent of its helicase activity and the cellular interferon pathway. Indeed, by its N-terminus, MOV10 binds to a protruding N-arm domain of N consisting of only 34 amino acids but proving important for N function and blocks N polymerization, N-RNA binding, and N-polymerase interaction, disabling RNP assembly. This study not only advances the understanding of bunyaviral replication and host restriction mechanisms but also presents novel paradigms for both direct antiviral action of MOV10 and host targeting of viral RNP machinery.

## Author summary

Severe fever with thrombocytopenia syndrome virus (SFTSV) is an emerging high-pathogenic bunyavirus listed by the World Health Organization as a top priority pathogen for research and development. Although SFTSV and related bunyaviruses emerging globally have raised serious public health concerns, specific antivirals or vaccines are currently unavailable and little is known on the virus-host interactions and viral replication mechanism. The nucleoprotein (N) is essential for bunyavirus replication by driving assembly of ribonucleoprotein (RNP), the RNA synthesis machinery and structural core of virions. Here we show that N proteins of SFTSV and related bunyaviruses can be targeted by host factor MOV10 in RNA-independent manner. Further, MOV10 can be induced specifically by the bunyaviruses and in turn restrict the viral replication and pathogenicity *in vitro* and *in vivo*. The anti-bunyavirus activity of MOV10 is independent of its helicase region and cellular interferon pathway. Instead, by its N-terminus, MOV10 binds to a protruding N-arm domain of N and blocks N polymerization, N-RNA binding, and N-polymerase interaction, disabling RNP assembly. This study provides a delicate model for host targeting of viral RNP machinery and sheds light on bunyaviral replication and host restriction mechanisms, which may promote specific antiviral therapy development.

## Introduction

The *Bunyavirales* order contains a large group of segmented negative-sense single-stranded RNA viruses, of which some members are significant pathogens causing severe diseases in humans and livestock [1]. Emerging tick-borne banyangviruses (classified as *Banyangvirus* genus in *Phenuiviridae* family of *Bunyavirales* order), including severe fever with thrombocytopenia syndrome virus (SFTSV) and Heartland virus (HRTV), have become new bunyavirus representatives highly pathogenic to humans [1, 2]. SFTSV firstly identified in China in 2009 and then reported in South Korea, Japan, and very recently, Vietnam, is the causative agent of the severe fever with thrombocytopenia syndrome (SFTS), characterized by fever, thrombocytopenia, elevated serum hepatic enzymes, gastrointestinal symptoms, hemorrhagic signs, and multiorgan dysfunction, and associated with high case fatality rates of up to 30% [3–7]. Due to the high pathogenicity, rapidly increasing incidence, expanding geographical distribution, and multiple transmission routes (including tick bites and human-to-human contacts), SFTSV was included by the World Health Organization (WHO) in the most dangerous pathogens requiring urgent research and development efforts. HRTV that can cause severe febrile illness in humans with clinical manifestations similar to SFTS was discovered in the United States in 2009 [8]. Following SFTSV and HRTV, Guertu virus (GTV) is the third representative species of *Banyangvirus* genus isolated from ticks collected in Xinjiang Province of China in 2014 [9]. Additionally, other emerging SFTSV-related viruses associated with febrile or uncharacterized illnesses continue to be reported around the world [10–12]. Together, as the new representatives of medically important bunyaviruses, SFTSV and the genetically related viruses have posed a severe threat to worldwide human health. However, there is currently no specific drug or vaccine available. Moreover, the knowledge of virus-host interactions and viral replication is quite limited, largely hampering the development of therapeutic and prophylactic strategies.

Similar to other bunyaviruses, SFTSV and related viruses contain three genomic segments, namely the large (L), medium (M), and small (S) segments [3]. The L and M segments encode the viral RNA-dependent RNA polymerase (RdRp, i.e. L protein) and the viral envelope glycoproteins (GP), respectively, and the S segment encodes the nucleocapsid protein (N) and a nonstructural protein (NSs) in an ambisense manner [3]. Previous studies by us and others have showed that the NSs proteins of banyangviruses play important roles at the interface of virus-host interactions by interfering with the interferon (IFN) antiviral immune responses [13–22], thus indirectly bolstering viral replication. However, NSs is neither the essential replication protein nor the structural component. In comparison, another viral protein encoded by the S segment, N, is directly required for RNA synthesis and virus propagation as the major structural element of both the viral ribonucleoprotein (RNP) and virion [23]. Multiple copies of N proteins that are polymerized encapsidate a viral RNA segment and associate with one copy of RdRp to form the RNP which acts as the RNA synthesis machinery in cytoplasm and also can be packaged by viral membrane and GP as the structural core of virions [23]. Structural studies revealed that as the building block of RNP assembly, banyangvirus N is composed of a compact core and a protruding N-terminal arm (N-arm) [24, 25]. The N-arm mediates N polymerization by binding to the neighboring N protomer, driving RNA encapsidation and RNP assembly [23, 25]. Considering the essential roles of N and the assembled RNP in virus replication and propagation, these fundamental viral elements, in turn, are likely vulnerable targets for host restriction of virus infection and further might be promising targets for antiviral intervention. However, it remains unclear which host proteins can target bunyavirus N and RNP and how the targeting affects bunyavirus infection and pathogenicity.

Here, we identified Moloney leukemia virus 10 protein (MOV10) as a host factor strongly targeting the N proteins of SFTSV (as the main model in this study) and related bunyaviruses. MOV10 that can be specifically induced by bunyaviruses in turn restricts the viral replication and pathogenicity *in vitro* and *in vivo*. A direct antiviral activity of host by targeting bunyavirus N actions to inhibit the viral RNP assembly is uncovered, which sheds lights on bunyavirus replication and host defense against bunyaviruses and provides new paradigms for MOV10 biological function and host interference with viral N/RNP machinery.

## Results

### Cellular protein MOV10 interacts with the N proteins of SFTSV and related bunyaviruses

To identify the potential host proteins binding to SFTSV N, HEK293 cells transfected with the plasmid encoding S-tagged N or the control vector were lysed for S-pulldown assays, followed by mass spectrometry analysis of the pulldown products. Interestingly, many peptides of MOV10 with high confidence were identified specifically in the N pulldown products but not in the control (S1 Fig). MOV10 has been shown to regulate replication of several viruses positively or negatively [26], although the underlying mechanisms are largely unclear. However, there is no report of MOV10 targeting of bunyaviruses. Thus, in this study, we investigated the role of MOV10 as a potential bunyavirus-targeting host factor.

**Fig 1:**
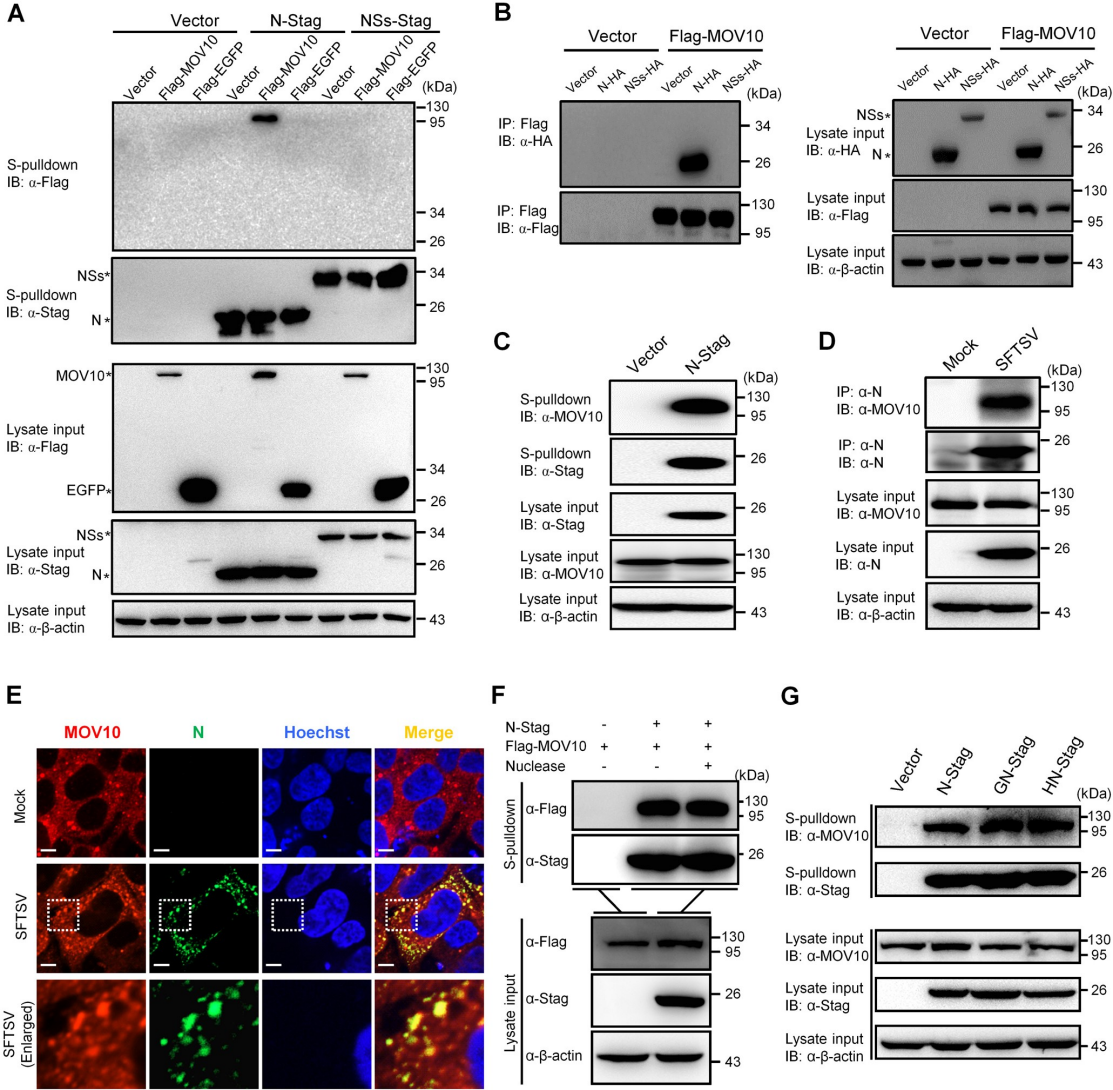
MOV10 strongly interacts with the N proteins of SFTSV and related bunyaviruses. (A) HEK293T cells were transfected with the plasmids encoding the indicated proteins, SFTSV N (N-Stag) or NSs (NSs-Stag) fused with S-tag or Flag-tagged MOV10 (Flag-MOV10) or EGFP (Flag-EGFP), or the corresponding control vectors. At 24 h posttransfection, the cells were lysed and subjected to S-pulldown and IB with the indicated antibodies, respectively. (B) HEK293T cells were transfected with the indicated protein expression plasmids or control vectors, followed by Co-IP assays (using the beads covalently conjugated with anti-Flag antibody) and IB analysis. (C) HEK293T cells transfected with the N-Stag expression plasmid or the control vector were respectively delivered to S-pulldown, followed by IB analysis. (D) HEK293T cells were mock infected or infected with SFTSV (MOI = 1) and then harvested for Co-IP and IB with the indicated antibodies. (E) Mock- or SFTSV-infected HEK293 cells were fixed for IFA at 24 h postinfection (hpi). Nuclei were stained with Hoechst. Scale bars, 8 μm. (F) HEK293T cells transfected with the indicated protein expression or control plasmids were lysed and then treated with excessive nuclease (Benzonase, Millipore, Cat#70746) or left untreated, followed by S-pulldown and IB. (G) HEK293T cells were transfected with plasmids encoding the indicated S-tagged N proteins of SFTSV (N-Stag), GTV (GN-Stag), or HRTV (HN-Stag) and then subjected to S-pulldown and IB as in (C). See also S1 and S2 Figs.

The interaction of MOV10 with SFTSV N was next validated by series of pulldown and Co-immunoprecipitation (Co-IP) assays combined with immunoblotting (IB) analyses. First, the IB analysis following S-pulldown assays showed that MOV10 could be specifically coprecipitated by SFTSV N (Fig 1A). Second, reciprocal interaction analysis by Co-IP with anti-Flag antibody covalently conjugated to magnetic beads consistently demonstrated the coprecipitation of SFTSV N with MOV10 (Fig 1B). Further, the strong interaction of SFTSV N with endogenous MOV10 was also confirmed by additional pulldown and Co-IP experiments in the contexts of transfection or SFTSV infection, respectively (Fig 1C and 1D). Then, cellular distribution of MOV10 and SFTSV N was monitored by immunofluorescence assay (IFA) and confocal microscopy. The results showed that N exhibits remarkable colocalization with MOV10 in cytoplasm upon SFTSV infection (Fig 1E), further confirming the association of N with MOV10. Considering the potential RNA-binding capacities of both MOV10 and N, we also tested the role of RNA in MOV10-N interaction. As shown in Fig 1F, nuclease treatment did not noticeably impair the co-precipitation of MOV10 with N, indicating that the MOV10-N interaction likely is not mediated by RNA. Additionally, endogenous MOV10 could be efficiently enriched in pulldown products of the N proteins encoded by HRTV and GTV as well, suggesting the strong interaction of MOV10 with these related N proteins (Fig 1G). Similarly, nuclease digestion could not abate the MOV10-N interactions either (S2 Fig). Together, these data establish that MOV10 is a cellular protein robustly interacting with the N proteins of SFTSV and related bunyaviruses likely independently of RNA.

### Induction of MOV10 expression by IFN and bunyaviruses

*MOV10* was included in previous studies and databases as a possible type I IFN-stimulated gene (ISG) [27–30]. However, published data characterizing the potential role of *MOV10* as an ISG is lacking. Therefore, we here tested type I IFN-stimulated expression of MOV10, in comparison with several typical ISGs. A series of human cells including HEK293, THP-1, A549, and HUVECs were used here for the analyses, as these cells derived from various tissue types were not only well-known to be sensitively stimulated by type I IFNs but also permissive for SFTSV infection (often used as cell models/targets for the viral infection) [18, 31, 32]. As expected, in HEK293 cells, IFN-α of various concentrations strongly triggered expression of all the well-known ISGs at 6 h posttreatment (S3A–S3C Fig); however, to our surprise, MOV10 expression was not significantly induced by the IFN even with high concentrations (S3D Fig). Consistently, time-course assays showed that MOV10 mRNA level was not evidently upregulated in the presence of IFN-α, in contrast to the rapid and substantial induction of the classical ISGs (S3E–S3H Fig). Similar experiments conducted with THP-1 monocytes showed that IFN treatment only triggered a weak and transient induction of MOV10, while expression of the well-known ISGs was greatly induced and still sustained at relatively high levels at 24 h after IFN stimulation (S4A–S4H Fig). Further, IFN stimulation of HUVEC and A549 cells also was shown to result in relatively weak induction of MOV10 (S4I–S4P Fig). Together, these data suggested that MOV10 cannot be efficiently (in HEK293) or only can be weakly (in other tested cells) induced by type I IFN, in comparison with the classical ISGs tested.

**Fig 2:**
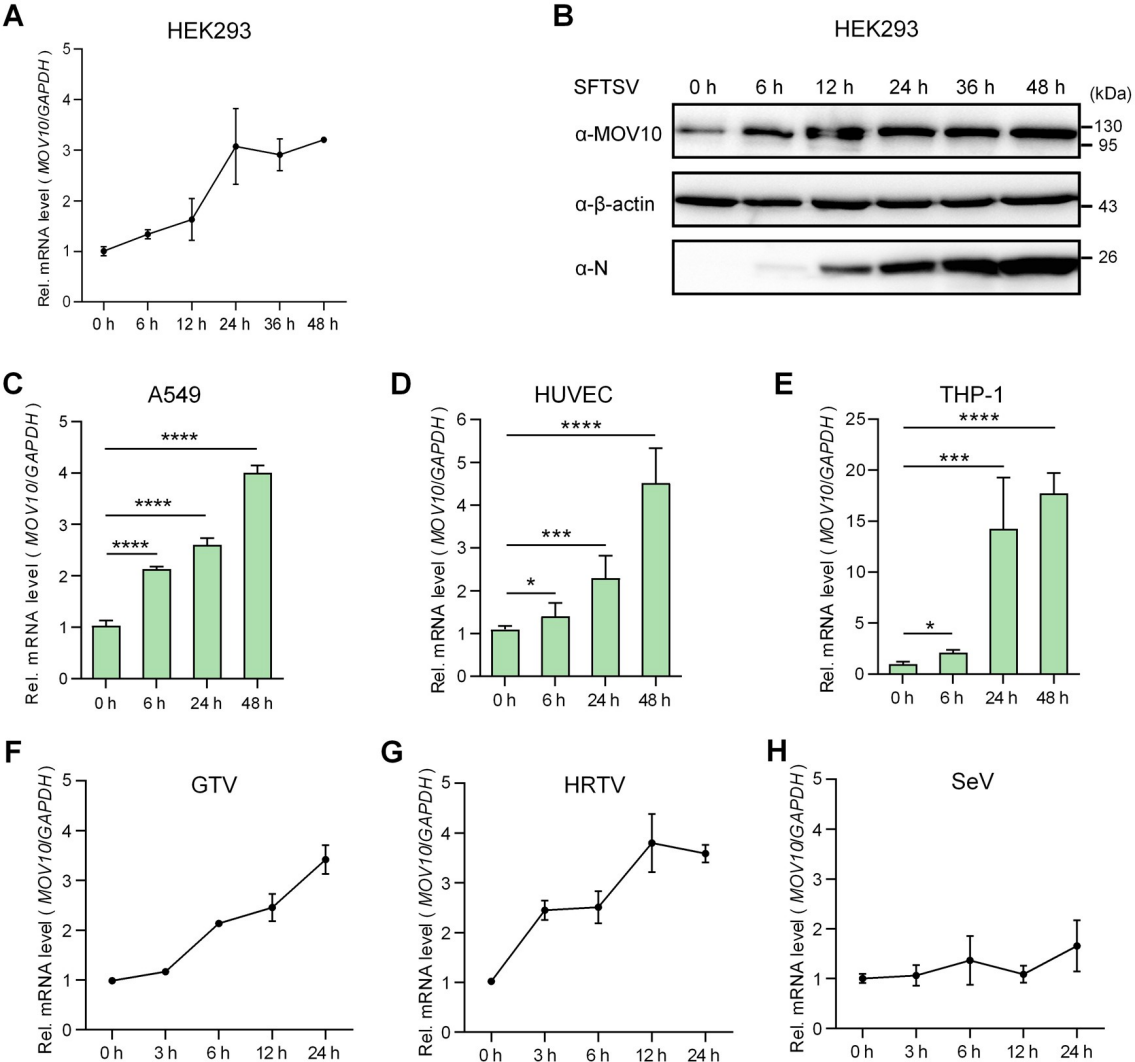
Specific upregulation of MOV10 expression by bunyavirus infection. (A-E) HEK293 (A and B), A549 (C), HUVEC (D), and THP-1 (E) cells were respectively infected with SFTSV (MOI = 5) and then harvested at the indicated time points (hpi) for evaluation of MOV10 mRNA levels by qPCR (A, C, D, and E) or protein levels by IB (B). (F-H) HEK293 cells infected with the indicated viruses, GTV (F), HRTV (G), or SeV (H), were delivered to analyses of MOV10 mRNA expression as performed in (A). Data show means ± standard deviations (SD), n ≥ 3. *p < 0.05; ***p < 0.001; ****p < 0.0001. See also S3 and S4 Figs.

To better characterize the potential role of MOV10 in bunyavirus infection, we next examined the expression of MOV10 upon SFTSV infection in the multiple human cells mentioned above. Interestingly, in contrast with the inability of IFN to induce MOV10 in HEK293, SFTSV infection obviously upregulated MOV10 expression in the human kidney cells (Fig 2A and 2B). Consistently, MOV10 expression also was stimulated by SFTSV infection in other tested human cells including A549, HUVEC, and THP-1 (Fig 2C–2E). Moreover, as indicated by the results obtained with THP-1, the induction of MOV10 by SFTSV seemed to be stronger and maintained at a high level even 48 hpi (~ 18 folds over the control) (Fig 2E), in comparison with the transient induction by IFN-α (S4H Fig). Similarly, both GTV and HRTV caused significant induction of MOV10 from early stages of infection as well (Fig 2F and 2G). However, by contrast, infection of Sendai virus (SeV, a model RNA virus) that resulted in dramatic upregulation of the typical ISGs (S3I–S3K Fig) did not evidently affect MOV10 expression (Fig 2H), reflecting the specific induction of MOV10 by the bunyaviruses.

Collectively, these data manifest that MOV10 can be specifically and sustainably upregulated by the bunyavirus infections, while its induction by type I IFN seems relatively weak, compared with the tested classical ISGs.

### MOV10 is a significant restriction factor of SFTSV and related bunyaviruses in infected cells

**Fig 3:**
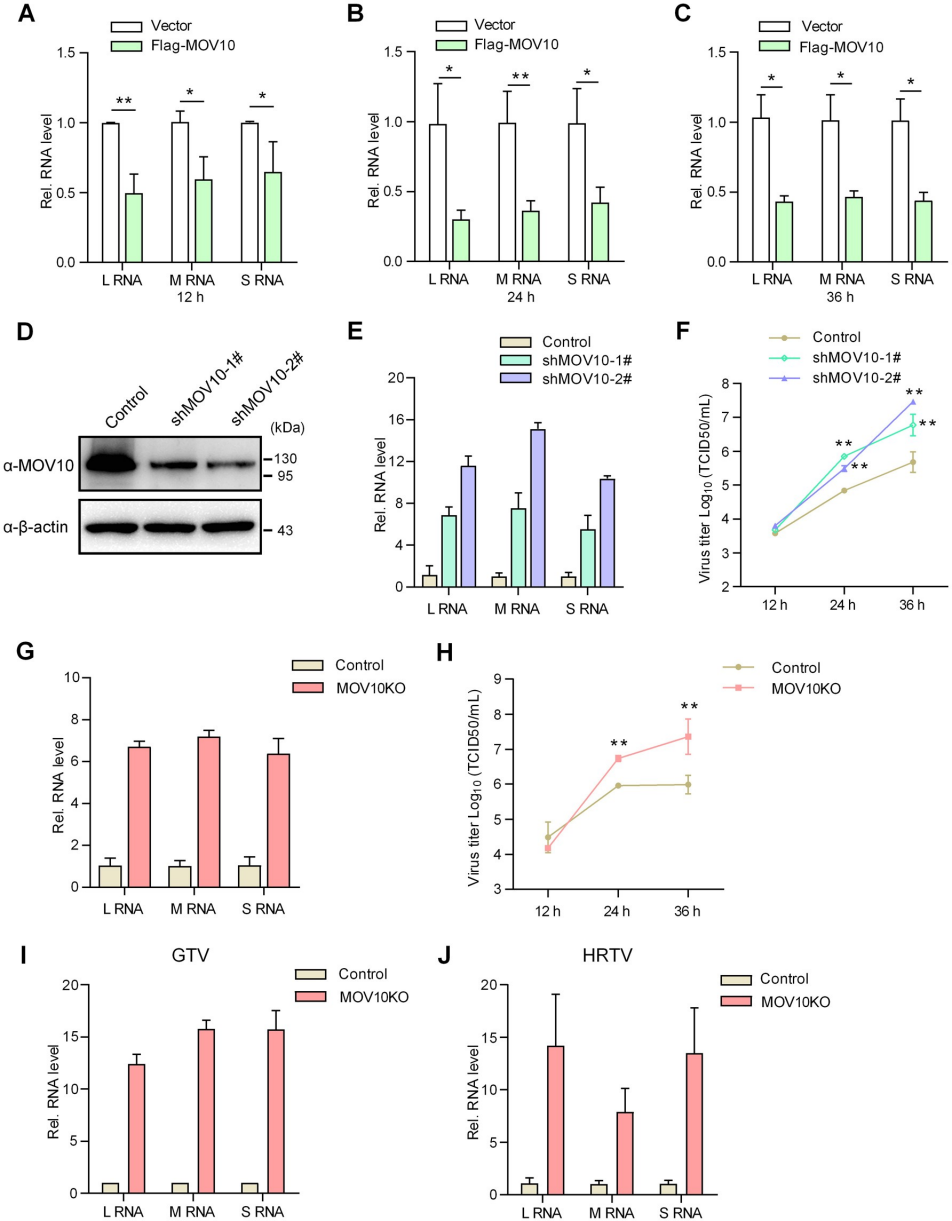
MOV10 inhibits replication of SFTSV and related viruses in infected cells. (A-C) HEK293T cells transfected with the Flag-MOV10 expression plasmid or control vector were infected with SFTSV (0.1 MOI) at 24 h posttransfection. At the indicated time points, replication levels of viral L, M, and S RNAs in infected cells were analyzed by qPCR. (D-F) HEK293T cells were transfected with the indicated shRNA-encoding or control plasmids for 48 h. Cells were then lysed for detecting MOV10 protein levels (D) or further infected with SFTSV (0.1 MOI) for analyzing viral RNA replication in infected cells at 36 hpi (E) or propagation of progeny viruses released into culture medium (F). (G and H) MOV10-KO or control HEK293T cells were infected with SFTSV (MOI = 0.1) and delivered to analyses of SFTSV RNA replication and progeny propagation as conducted in (E and F). (I and J) MOV10-KO or control cells were infected with GTV (I) or HRTV (J) at MOI of 0.1 and delivered to analyses of virus RNA replication as conducted in (G). In these qPCR analyses, relative RNA levels over the control groups (wildtype cells or the cells transfected with the control plasmids) were calculated for each segment, respectively. Data show means ± SD, n ≥ 3. *p < 0.05; **p < 0.01. See also S5A and S5B Fig.

N plays a core role in bunyavirus life cycle [23]. Given the strong interaction of MOV10 with N and cellular upregulation of MOV10 upon bunyavirus infection, we considered that MOV10 might modulate the bunyavirus replication. Indeed, as shown in Fig 3A–3C, overexpression of MOV10 significantly reduced SFTSV RNA replication. Conversely, knockdown (KD) of MOV10 by shRNA-mediated RNAi evidently augmented the virus RNA replication (Fig 3D and 3E). Moreover, virus growth curves showed that MOV10 KD remarkably bolsters the propagation of progeny SFTSV (Fig 3F). Further, we evaluated SFTSV replication in MOV10-knockout (MOV10-KO) cells generated by CRSPR-Cas9 system (S5A and S5B Fig). Consistently, MOV10 KO resulted in pronounced enhancements of SFTSV RNA replication and progeny propagation (Fig 3G and 3H). Together, these results demonstrate that MOV10 is a notable host restriction factor of SFTSV in infected cells.

Additionally, replication of GTV and HRTV was also analyzed in the MOV10-KO and control cells by qPCR. Similarly, MOV10 deletion resulted in dramatic increase of both GTV and HRTV RNA levels (Fig 3I and 3J). These data further confirm the remarkable role of MOV10 as a robust antiviral factor of host cells against SFTSV and related bunyaviruses.

### Knockdown of MOV10 renders mice more susceptible to SFTSV infection

**Fig 4:**
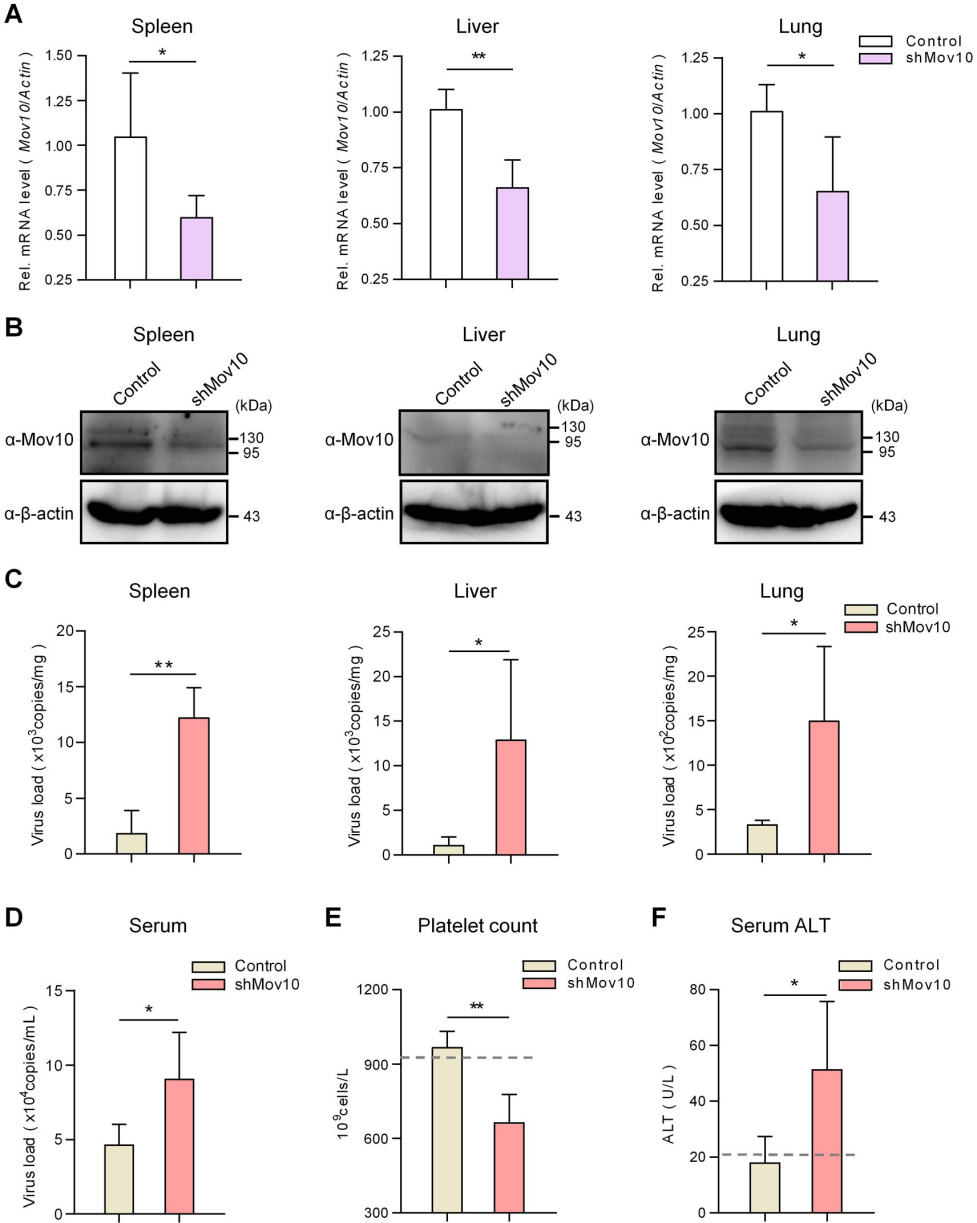
MOV10 restricts SFTSV infection and pathogenicity *in vivo*. C57/BL6 mice (n = 5/group) were transduced with the viral vectors encoding control or Mov10-targeting shRNAs (5×10^7^ TU) via caudal vein, followed by infection with SFTSV (10^5^ TCID50) via subcutaneous injection, 7 d posttransduction. Three days postinfection, mice were sacrificed for evaluation of Mov10 expression by qPCR (A) and IB (B) analyses and for quantification of viral S RNA copy (C and D), platelet count (E), and ALT level (F) in the indicated tissues as described in Materials and Methods. Data are means ± SD. *p < 0.05; **p < 0.01. Dotted lines represent the average platelet count (E) or ALT level (F) of control mice without transduction or infection (n = 5) for reference. See also S6 Fig.

Adult mice could be infected by SFTSV with detectable virus in various organs, although the infection in the adult animals was transient without noticeable clinical features [33]. Since MOV10 showed remarkable anti-bunyavirus activity in cultured cells, we asked whether MOV10 affects the virus infection and pathogenicity *in vivo*. As MOV10 is essential for neuronal development in mice and its knockout is embryonic lethal [34], we here exploited a MOV10-KD mouse model by transient transduction of lentiviral vectors expressing specific shRNA via intravenous injection. Adult mice were transduced with the control or Mov10-targeting shRNA expression vectors, followed by SFTSV challenge at 1 week posttransduction. No severe clinical manifestations or deaths were found in all infected animals (n = 5 per group) in the following days of continual observation. Thus, to more closely monitor the effects of Mov10 KD, infected mice were sacrificed for further analyses in the repeated experiment. qPCR and IB suggested that Mov10 indeed was partially silenced by the specific shRNA in tested mouse organs (Fig 4A and 4B). Importantly, viral RNA copies in the organs of mice with Mov10 KD were remarkably increased compared to the control (Fig 4C). Moreover, the serum viral load in Mov10-silenced mice was also significantly higher than the control (Fig 4D), indicating an elevated viremia due to Mov10 KD. These findings indicate that MOV10 downregulation boosts SFTSV replication *in vivo*, consistent with the data obtained with cultured cells. Furthermore, in line with the enhanced virus replication, we detected that SFTSV infection in the Mov10-KD animals (but not the control) significantly induced platelet count reduction (Fig 4E) and alanine transaminase (ALT) level increase (an indicator of liver dysfunction) (Fig 4F), which both are major clinical features of human infection with SFTSV. In addition, we did not observe noticeable influence of viral transduction itself on the viral infection in mice, compared with non-transduced animals (S6 Fig). Together, these findings suggest that MOV10 likely also contributes to host restriction of SFTSV replication and pathogenicity *in vivo*.

### MOV10 targets SFTSV RNP machinery to inhibit virus replication independently of IFN signaling

**Fig 5:**
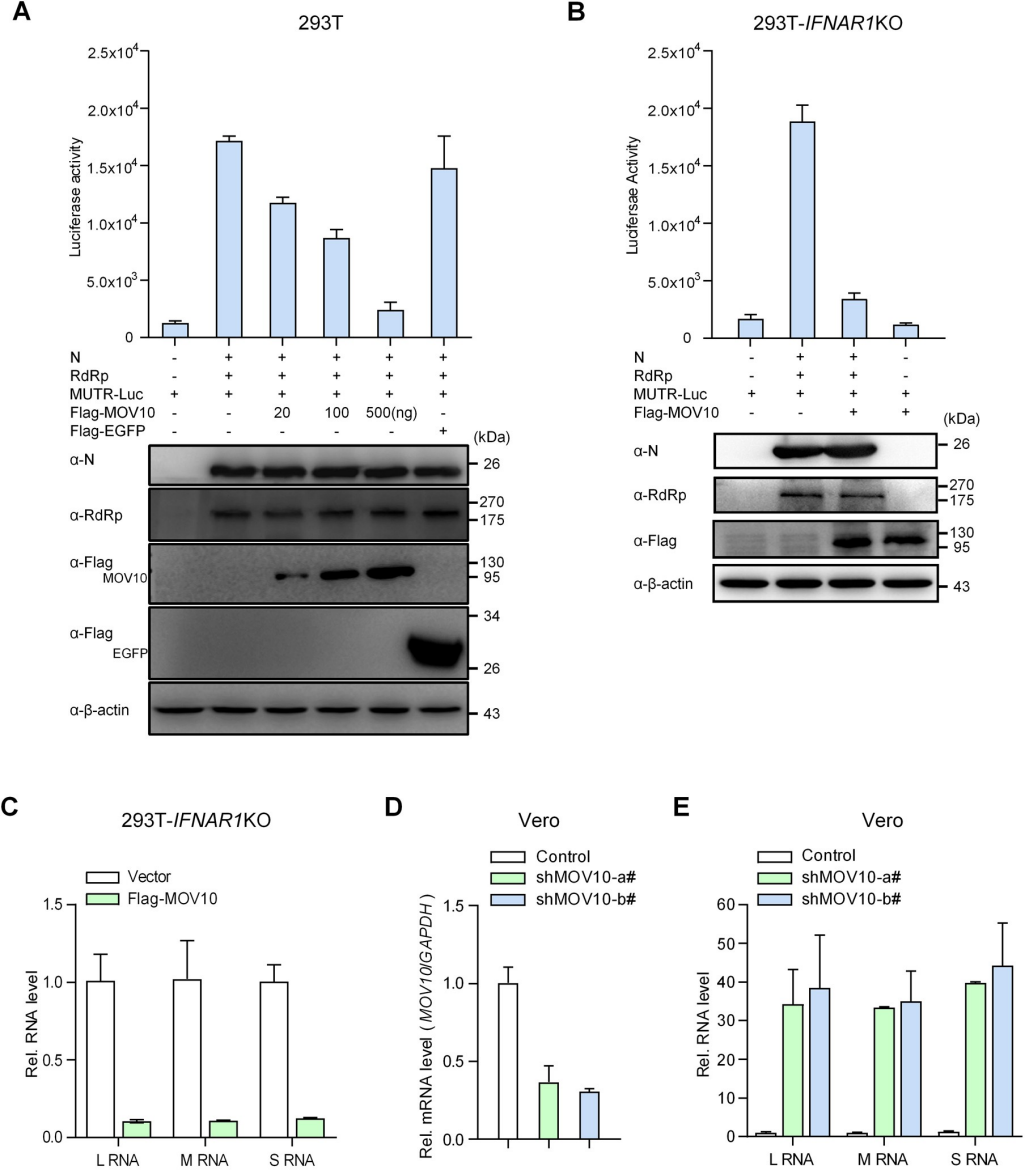
MOV10 targets RNP machinery to inhibit virus replication independently of IFN signaling. (A) HEK293T cells were cotransfected with SFTSV RdRp and N expression plasmids and the plasmid producing the M RNA analogue containing negative-sense firefly luciferase gene sequence (MUTR-Luc), together with increasing amounts of the Flag-MOV10 expression plasmid or 500 ng of the Flag-EGFP plasmid. At 48 h posttransfection, cells were delivered to luciferase activity measurement and IB detection. (B) IFN receptor-KO HEK293T cells generated by CRISPR-Cas9 were used to assess MOV10 suppression of SFTSV RNP by the RNP reconstitution minigenome system as in (A). (C) IFN receptor-KO HEK293T cells transfected with the control or MOV10 expression plasmids were infected with SFTSV (0.1 MOI), followed by evaluation of virus RNA replication by qPCR at 24 hpi. (D and E) Vero cells were transduced with the lentiviral vectors expressing control or MOV10-targeting shRNAs. At 48 h posttransduction, cells were infected with SFTSV (0.1 MOI), followed by detection of KD efficiency (D) and viral RNA replication (E) at 24 hpi with qPCR. Data show means ± SD, n = 3. See also S5C, S5D, S7A, and S7B Figs.

Bunyavirus RNA synthesis takes place in the context of the RNP machinery that is comprised of one of the virus RNA segments, polymerized N proteins coating the RNA segment, and one molecule of RdRp by interactions of these components [23]. Because of the strong interaction of MOV10 with N and the essential role of N in virus infection as the main RNP component, we considered that by targeting N, MOV10 may interfere with RNP and hence virus replication. To validate the consideration, we tested the effect of MOV10 on SFTSV RNP activity using a minigenome reporter system that constructs the RNP machinery for RNA synthesis in cells. As shown in Fig 5A, the reporter gene (firefly luciferase) was successfully activated in the presence of N and RdRp expression, indicating the efficient RNP assembly and functioning. Interestingly, MOV10 expression indeed suppressed the RNP-driven reporter activity in a dose-dependent manner (Fig 5A). We also employed a dual-luciferase reporter system for the minigenome assay by including an additional control plasmid expressing *Renilla* luciferase that showed consistent results for the dose-dependent inhibition of the RNP-driven reporter activity (S7A Fig). These data suggest that in line with its targeting of N, MOV10 can interfere with assembly of RNP or working of assembled RNP on RNA synthesis.

Many host factors can attenuate viral replication indirectly by modulating cellular IFN system, while targeting of N and inhibition of RNP system by MOV10 support that MOV10 likely has direct anti-bunyavirus ability. To determine whether MOV10 can inhibit bunyavirus replication directly by targeting N and hence RNP, but not by regulating IFN response, we assessed the capacity of MOV10 to block RNP activity and viral replication in the contexts of IFN pathway deficiency. Firstly, in IFN receptor (IFNAR1)-deleting cells generated by CRISPR-Cas9 editing (S5C and S5D Fig), minigenome assays showed that MOV10 still can greatly abolish SFTSV RNP activity (Figs 5B and S7B). Moreover, disruption of IFN signaling did not impair the potent inhibition of SFTSV RNA replication by MOV10 (Fig 5C). Further, Vero cells that are defective in IFN production were also used to analyze the anti-bunyavirus activity of MOV10. Endogenous MOV10 KD in Vero cells led to dramatic increase of SFTSV RNA replication, revealing the robust anti-SFTSV capability of MOV10 in absence of IFN production (Fig 5D and 5E). Collectively, these observations suggest that MOV10 can strongly suppress SFTSV RNP formation or action and hence RNA replication independently of the IFN pathway, further supporting the direct anti-bunyavirus ability of MOV10.

### MOV10 targets N and represses RNP machinery by its N-terminal domain, while its RNA helicase region is dispensable

**Fig 6:**
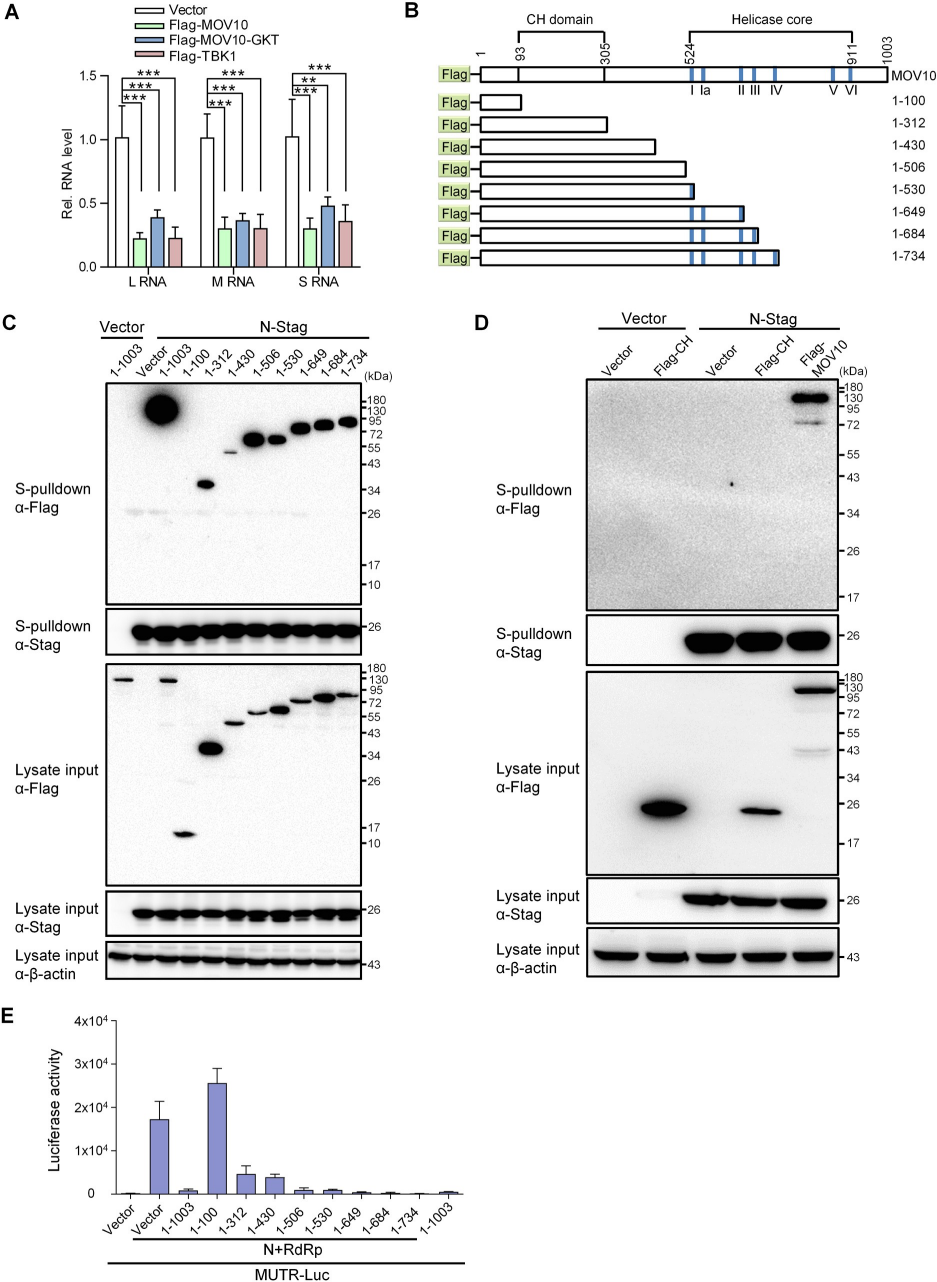
The N terminal domain (but not helicase region) of MOV10 is required for its targeting of N and inhibition of RNP. (A) HEK293T transfected with the control vector or the plasmids expressing Flag-tagged MOV10, MOV10GKT (helicase-inactive mutant with G529A/K530A/T531A substitutions), or TBK1 were infected with SFTSV (0.1 MOI), followed by detection of virus RNA levels at 24 hpi with qPCR. (B) Schematic diagrams showing the organization of Flag-tagged MOV10 and truncated mutants used below. The helicase core contains 7 motifs, i.e., I, Ia, II, III, IV, V, and VI. (C and D) HEK293T cells co-transfected with the plasmids encoding the indicated proteins were subjected to S-pulldown assays, followed by IB detection. (E) Effects of the truncated MOV10 mutants on SFTSV RNP activity were analyzed with the minigenome system as performed in Fig 5A. Data show means ± SD, n = 3. **p < 0.01; ***p < 0.001. See also S7C Fig.

MOV10 is a putative RNA helicase and the potential helicase activity is likely important for its physiological function and previously reported antiviral activity [35–38]. To test the role of MOV10 helicase activity in its anti-bunyavirus effect, we first compared the anti-SFTSV capabilities of wildtype MOV10 and the helicase activity-deficient mutant (MOV10-GKT) [38]. In conformity with the results above, wildtype MOV10 caused a strong inhibition of SFTSV RNA replication, which is comparable to that mediated by TBK1 (Fig 6A), a pivotal kinase of antiviral immunity [18]. Meanwhile, the mutant with helicase activity disrupted appeared to retain remarkable anti-SFTSV effect (Fig 6A), indicating that the helicase activity is not essential for MOV10 inhibition of SFTSV. Further, we investigated the domain(s) of MOV10 required for the targeting of N and the anti-bunyavirus activity using a series of truncated MOV10 mutants (Fig 6B). Pulldown assays demonstrated that a truncated mutant consisting of only the N-terminal 1–312 aa and the other longer mutants which contain the region of 1–312 aa all exhibit evident interaction with N, whereas a shorter form (aa 1–100) cannot be coprecipitated with N (Fig 6C). Furthermore, the Cys-His-rich (CH) domain alone was not pulled down with N either (Fig 6D). As the helicase motifs were located in C-terminus (524–912 aa) (Fig 6B), these results indicate that the N-terminus (aa 1–312) is the N-binding region, whereas the helicase activity and helicase domain are dispensable for MOV10 interaction with N. Additionally, the results are in agreement with the observation of RNA-independent MOV10-N interaction, because the helicase core also is responsible for RNA binding [38]. In accordance with the activity to target to N, the truncated proteins that can interact with N preserved inhibitory effects on SFTSV RNP, while the mutant consisting of 1–100 aa that loses the ability to bind to N did not (Figs 6E and S7C). The consistency between the capabilities of interaction with N and inhibition of RNP further links MOV10 targeting of N with its anti-bunyavirus activity. Taken together, the data suggest that MOV10 inhibits RNP and hence virus replication by the N terminal domain through targeting of N, while its helicase region (as well as the related functions like the RNA binding and helicase activities) is not necessary for the interaction with N and the anti-bunyavirus activity.

### MOV10 impairs N oligomerization and N interactions with RNA and RdRp by binding to the N-arm domain of N, inhibiting RNP assembly

**Fig 7:**
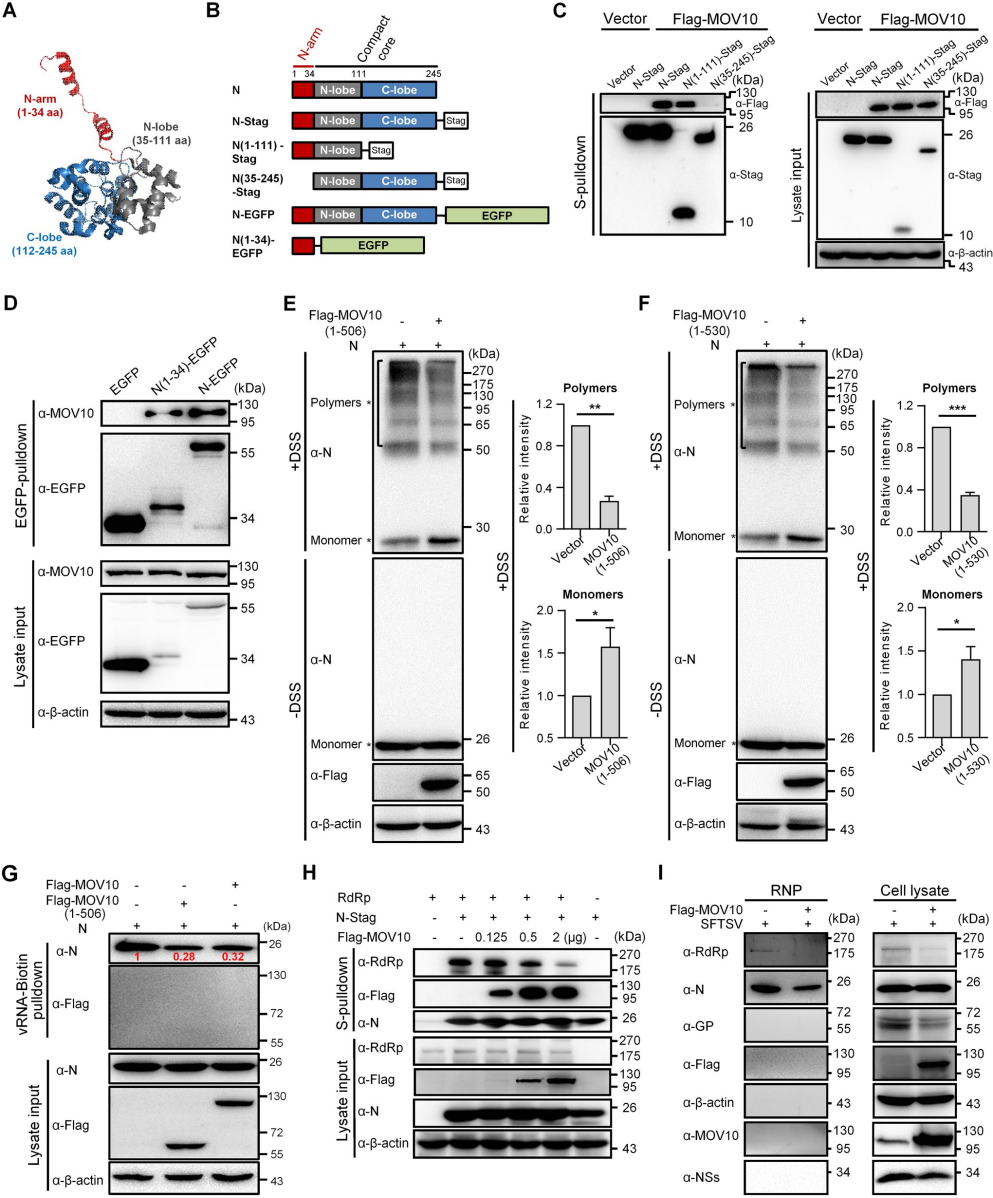
By binding to the N-arm of N, MOV10 blocks N polymerization, N-RNA binding, and N-RdRp interaction, inhibiting RNP assembly. (A) The structure of SFTSV N monomer (PDB: 4J4R). SFTSV N monomer folds into a compact core with a protruding N-arm. The core further consists of the N- and C-lobes. (B) Schematic diagram of N truncations used below. (C) HEK293T cells were cotransfected with the plasmids expressing S-tagged N mutants and Flag-MOV10 or the control vectors for 24 h and then subjected to S-pulldown and IB analyses. (D) HEK293T cells transfected with the plasmids encoding EGFP or EGFP-tagged proteins were subjected to EGFP-nanotrap and IB. (E and F) MOV10-KO HEK293T cells were transfected with the Flag-tagged MOV10(1–506) or MOV10(1–530) expression plasmids or control vector, together with the expression plasmid for SFTSV N, and cross-linked with disuccinimidyl suberate (DSS) that enables the fixation of oligomerized bunyavirus N *in situ*, followed by SDS-PAGE and IB analyses. Asterisks indicate N monomers and polymers based on the apparent molecular weights. The band intensities of polymers and monomers of the DSS-treated samples were quantified using ImageJ software and then the relative intensities (over the controls without MOV10(1–506) or MOV10(1–530) expression) were respectively calculated and shown in the graphs beside the representative IB results. Data are means ± SD, n = 3. (G) HEK293T cells were transfected with the indicated protein expression plasmids before the pulldown assay using biotin-labeled *in vitro* transcript of SFTSV RNA and streptavidin-coupled beads. The cell lysate inputs and pulldown products were then analyzed by IB. Band intensities of RNA-bound N were quantitatively measured with the ImageJ software and indicated in the blot after normalization to the control without Flag-MOV10 or Flag-MOV10(1–506) expression. (H) HEK293T cells were transfected with plasmids encoding RdRp and N-stag, together with increasing amounts of Flag-MOV10 expression plasmid, and then subjected to S-pulldown and IB. (I) HEK293 cells transfected with Flag-MOV10 or vector were infected with SFTSV (3 MOI), followed by extraction of RNP from cell lysates at 24 hpi. The cell lysates and extracted RNP were respectively subjected to IB with the indicated antibodies. See also S8, S9 and S10 Figs.

To further unravel the mechanism underlying MOV10 targeting of N, we next mapped the domain of N interacting with MOV10, using truncated N mutants (Fig 7A and 7B). In S-pulldown assays, the S-tagged mutant with C-lobe of the core domain deleted, ie. N(1–111)-Stag, but not the N-arm-deleting mutant N(35–246)-Stag, could pull down MOV10 efficiently (Fig 7C), revealing that the N-arm is required for N-MOV10 interaction. Interestingly, by an EGFP-nanotrap assay, we further demonstrated that MOV10 was coprecipitated specifically with the EGFP-tagged N-arm but not the EGFP control (Fig 7D), indicating that the N-arm is likely sufficient to mediated the interaction with MOV10. Together, these results suggested that the N-arm containing only 34 aa is the MOV10-interacting domain of N.

Given the essential role of the N-arm in N polymerization, we considered that the interaction of MOV10 with N-arm might obstruct N polymerization and hence RNP assembly. To confirm the notion, the effect of MOV10 on formation of N polymers was examined by chemical cross-linking assays combined with electrophoresis separation under denaturing conditions. Here, MOV10(1–506) which was shown to interact intensively with N and harbor the antiviral activity comparable with that of the full-length protein (Fig 6) was first used to avoid influences of the large helicase region of MOV10 and its RNA-binding activity on the cross-linking and N oligomerization analyses. As shown in Fig 7E, without cross-linking, SFTSV N migrated as a monomer under the denaturing conditions of SDS-PAGE. In comparison, after the cross-linker treatment, various species of N oligomers were revealed and correspondingly, the monomer was reduced in band intensity (Fig 7E). However, in the presence of MOV10(1–506), the polymeric N proteins were decreased and meanwhile the monomer was accordingly increased, compared to the control without MOV10(1–506) expression (Fig 7E), indicating that MOV10(1–506) interferes with N polymerization, consistent with the targeting of N N-arm. Similarly, inhibitory effect of MOV10(1–530) on N polymerization was also observed: expression of the protein resulted in loss of N polymers, accompanied by increase of the monomer (Fig 7F). Consistent with the inability of MOV10(1–100) to interact with N (Fig 6C) or to interfere with RNP construction (Fig 6E), this MOV10 mutant, by contrast, did not exhibit noticeable influence on N polymerization (S8 Fig). These findings suggest that MOV10 can block N polymerization, the fundamental requirement for RNP assembly, by interacting with the N-arm of N.

In an active bunyavirus RNP, polymerized N proteins bind to viral segment RNA for encapsidation that not only can protect the virus genome but also is essential for RNP formation and functioning on facilitating RdRp-catalyzed RNA synthesis [23]. Previous data also showed that SFTSV N oligomerization mediated by N-arm is likely necessary for RNA binding of N [25]. Considering the inhibition of N oligomerization by MOV10 targeting of N-arm, we investigated whether N binding with virus RNA thus can be impaired by MOV10. As shown in SFTSV RNA-pulldown assays, binding of N with virus RNA indeed was diminished by the expression of MOV10 or MOV10(1–506) (Fig 7G), in line with the MOV10 blockade of N oligomerization. Meanwhile, unlike N, MOV10 did not efficiently bind to the virus RNA (Figs 7G and S9), which consists with the RNA-binding specificity of MOV10 [38] and rules out the competitive RNA binding between N and MOV10. As N polymerization, N-RNA binding, and N interaction with RdRp are all important for functional assembly of RNP, we further analyzed whether MOV10 targeting of N also affects the interaction of N with RdRp. As indicated in Fig 7H, the interaction between N and RdRp indeed was dose-dependently inhibited by MOV10 as well. These data suggest that apart from the blocking of N polymerization, MOV10 is likely able to interfere with N-RNA binding and N-RdRP interaction as well, which would further contribute to the inhibition of RNP formation.

Consistent with the findings above, production of RNPs in MOV10-overexpressing cells was reduced as suggested by IB analysis of extracted SFTSV RNPs: the N as well as RdRp packaged into RNPs was obviously less in the context of MOV10 overexpression (Fig 7I). In addition, we noted that there was no visible endogenous or overexpressed MOV10 detected in the RNPs (Fig 7I); moreover, MOV10 was not detected in purified SFTSV virions either (S10A–S10C Fig), suggesting that MOV10 cannot interact with the already formed RNPs. This is consistent with the likely competitive binding of MOV10 to N N-arm that hinders N polymerization also requiring the N-arm domain. Consequently, we considered that MOV10 is likely unable to interfere with the assembled RNP functioning. To validate the consideration, we tested the effect of MOV10 on functioning of the formed RNP using infection assays with an infectious virus-like particle (iVLP) in which assembled RNP is further packaged by SFTSV GP. As expected, MOV10 did not significantly block the reporter activity mediated by the formed RNP brought by the incoming iVLP (S10D Fig), corroborating that MOV10 cannot affect the action of assembled RNP.

Altogether, these data suggest that MOV10 inhibits RNP assembly (but not assembled RNP functioning) by interacting with the N-arm of N and thus blocking N polymerization, N-RNA binding, and N-RdRp interaction, leading to the restriction of bunyavirus replication and pathogenicity.

## Discussion

**Fig 8:**
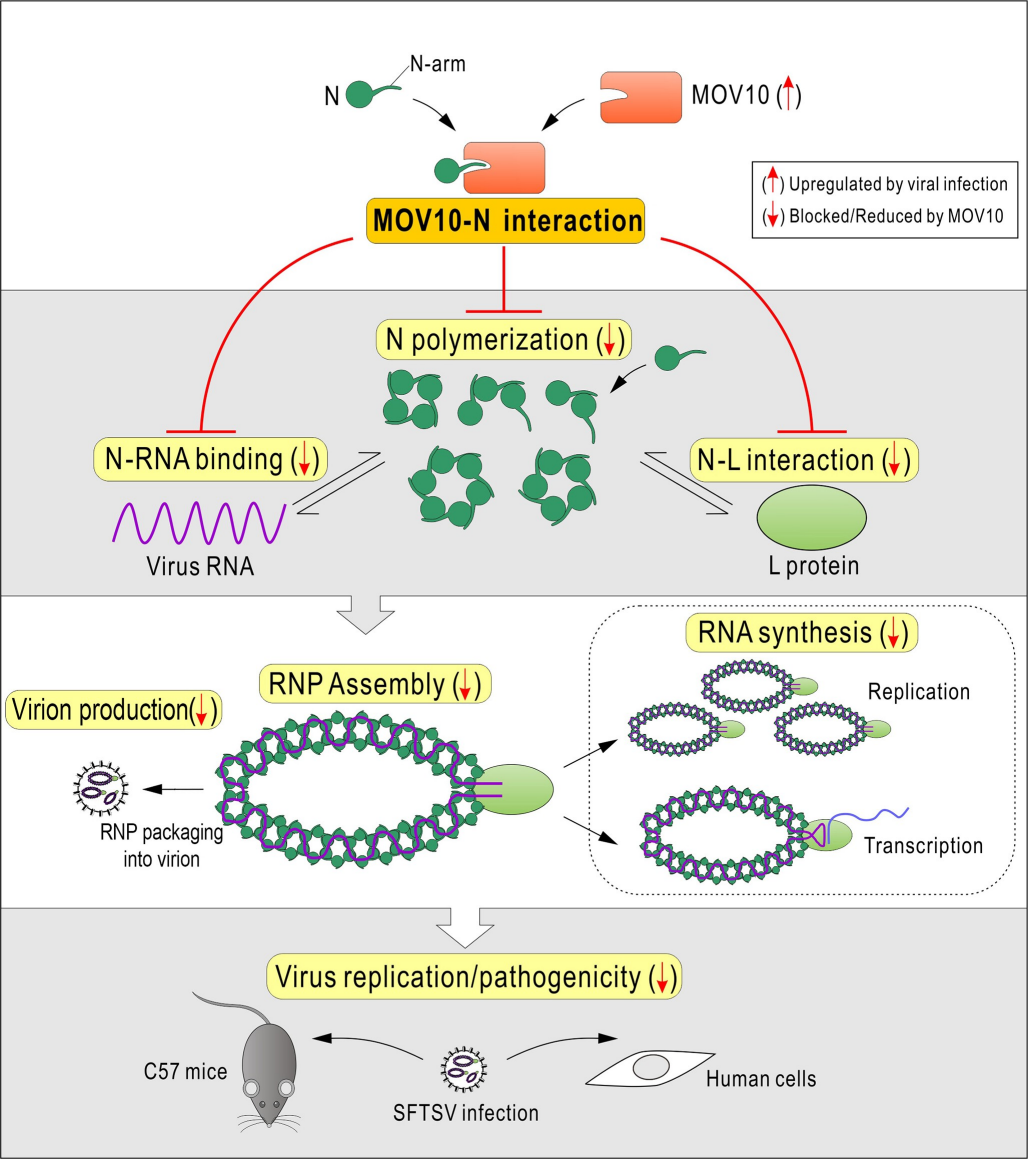
Model for host restriction of bunyaviruses via MOV10 by targeting N to block RNP assembly.

SFTSV and related bunyaviruses discovered globally have raised serious public health concerns worldwide. It is urgently needed to understand the virus-host interactions and viral replication mechanism for antiviral therapeutic development. Given the central roles of N and N-assembled RNP in bunyaviral replication, we considered that they could be assailable targets for host defense, although it was unknown which and how host factors target the N and RNP of these high-pathogenic bunyaviruses before the present study. Here, we identified MOV10 as a robust banyangvirus N-interacting protein and as a remarkable host restriction factor of the bunyavirus replication and pathogenicity by *in vitro* and *in vivo* experiments. Moreover, we found that although MOV10 is a weak ISG, it can be significantly and specifically induced in response to infections of SFTSV and related bunyaviruses, likely serving as an add-on to the anti-bunyavirus function of MOV10. By RNA-independent targeting of N and inhibition of RNP, MOV10 exhibits a direct anti-bunyavirus activity, which is independent of the cellular IFN response. The N-terminal domain but not the large helicase region then was shown to be required for the anti-bunyavirus action of MOV10. Furthermore, MOV10 binds to the N-arm of N and blocks N polymerization, N-RNA binding, and N-RdRp interaction, disabling the formation of RNP machinery and restricting the bunyavirus replication and pathogenicity (summarized in a proposed model, Fig 8). This study demonstrates a clear and novel mechanism for host restriction of bunyavirus replication and pathogenicity through artful targeting of N/RNP machinery by MOV10, providing valuable insights into bunyavirus-host interactions. To our knowledge, this is the first to elucidate host targeting of the N/RNP of these life-threatening bunyaviruses, understanding of which may not only shed lights on the mechanisms of host defense and viral replication but also promote the design of antiviral therapeutics in the future.

MOV10 is a putative RNA helicase that was shown to have 5' to 3' directional helicase activity *in vitro* and likely be involved in UPF1-regulated RNA transcript metabolism [38]. MOV10 was also shown to associate with argonaute-2, a protein involved in miRNA-mediated gene regulation, in RNA-dependent manner, although it remains uncertain whether MOV10 plays a role in miRNA modulation [39, 40]. Additionally, in neurons, MOV10 likely regulates translation of some NMDAR-responsive mRNAs and is essential for normal neuronal development [34, 41, 42]. It should be noted that these actions of MOV10 are dependent on its RNA-binding or helicase activities [38, 40, 42]. Interestingly, MOV10 inhibits bunyavirus replication independently of the RNA-binding or helicase activities, indicating that MOV10 has the unique anti-bunyavirus capacity not involving the potential physiological actions above. It is consistent with the direct anti-bunyavirus activity of MOV10 by targeting N and RNP discovered here. Additionally, we demonstrated that MOV10 retains its robust anti-bunyavirus capability in the IFN system-deficient contexts, also in line with its direct inhibitory activity against bunyaviruses, although MOV10 and IFN responses would be likely to restrict viral replication synergistically during systematic infection [27, 43]. It might be noted that the direct antiviral activity of MOV10 here was established mainly based on the data obtained in cell models, while during systematic infection *in vivo*, some potential, indirect effect of a host antiviral factor on virus-host interactions might also contribute to the antiviral responses. Thus, besides the direct anti-bunyavirus activity, whether other effects of MOV10, especially its potential roles in host physiological processes (including those unknown), are also somehow involved in the antiviral defenses during systematic infection may need to be further explored in the future.

Previously, MOV10 was referred as a possible ISG and the evidence was from some microarray analysis [27–30]. However, there were no published experimental data detailedly characterizing its induction by IFN. Here, our study not only demonstrates the expression kinetics and the limited inducibility of MOV10 upon IFN treatment but also suggests that MOV10 is a gene specifically stimulated by banyangviruses. Upregulation of MOV10 by the bunyaviruses appeared to be more sustainable, compared to the weak and transient stimulation by IFN. The induction of MOV10 in host cells could act as amplifier of its anti-bunyavirus effect, contributing to host restriction of the bunyaviruses. It will be interesting to further delineate the mechanism for the specific upregulation of MOV10 in response to SFTSV and the related bunyavirus infections.

There are previous studies reporting that MOV10 could play potential roles in modulating replication of several viruses [26]; however, the corresponding mechanisms are largely obscure. For instance, MOV10 KD assays showed that MOV10 could restrict the replication of Dengue virus [44], but is likely required for hepatitis D virus replication [45], while the underlying mechanisms remain unclear. MOV10 was also shown to be a possible restriction factor of influenza A virus (IAV) [46], which will be further discussed below. Additionally, MOV10 seems to influence the replication of retroviruses and retroelements [26, 37, 47], but a study on endogenous MOV10 reported that endogenous MOV10 inhibits endogenous retroelements but has no effect on the replication of exogenous retroviruses [39]. These reports suggest that the roles of MOV10 as a potential regulator of the viral infections may be negative or positive depending on the specific viruses or expression contexts and need more investigations to be functionally and mechanistically determined. In our study, loss- and gain-of-function experiments gave the consistent conclusion for MOV10 inhibition of SFTSV and related bunyaviruses in infected cells. Moreover, there was no report referring to the possible function of MOV10 in modulating viral replication *in vivo* previously, while here functional analyses using a MOV10-KD animal model also supported the anti-bunyavirus role of MOV10. Furthermore, the anti-bunyavirus mechanism of MOV10 by targeting N and blocking RNP assembly was detailedly unraveled. Consequently, this study provides a clear example for the direct antiviral activity of MOV10, expanding the knowledge of MOV10 biology. However, it should be noted that the N proteins of SFTSV and related bunyaviruses are conserved in *Banyangvirus* but share low or no noticeable sequence identity with other bunyaviruses out of the genus [3, 8, 9]. Thus, whether and how MOV10 has inhibitory effect on phleboviruses (*Phlebovirus* genus, the most related genus with *Banyangvirus* in *Phenuiviridae* family of *Bunyavirales* order) and other more distant bunyaviruses needs to be further investigated, while our study here may present some instructive information and methods for such future investigations.

Due to the essential role of MOV10 in development, we employed an adult animal model with MOV10 KD to evaluate the anti-bunyavirus effect *in vivo*. Indeed, MOV10 KD resulted in increased viral replication and viremia, accompanying with platelet count reduction and liver injury marker release in serum. The data suggest that MOV10 likely plays a role in restricting viral replication and pathogenicity *in vivo*, further supporting the antiviral effect of MOV10 observed in cultured cells. However, MOV10 KD in the animal model is transient and partial and thus the antiviral role of MOV10 could be significantly underestimated therein. Moreover, adult animals are generally high resistant to these virus infections [33]. It will be merited to explore other strategies or other animals perhaps more susceptible, such as cats or aged ferrets reported very recently [48, 49], for loss-of-function investigation to better assess the role of MOV10 in host antiviral defense *in vivo*.

Since N-assembled RNPs are the viral RNA synthesis machineries and the main structural components of virions, it is reasonable to speculate that RNPs of viruses thus could be targets of host restriction. An extensively investigated example is the RNP of influenza virus. Several host proteins have been shown to inhibit IAV RNP by interacting with the RNP components, including NP (the nucleoprotein of IAV), polymerase subunits, or RNA [46, 50–56]. Particularly, TRIM25, a cellular E3 ubiquitin ligase, was shown to bind IAV RNP in RNA-dependent manner, inhibiting RNA synthesis [55]. Although the exact RNP protein component targeted by TRIM25 is unknown, the authors conjectured that TRIM25 might interact with IAV NP in the context of the RNP complex [55]. In addition, coincidently, MOV10 was also shown to bind IAV RNP by interacting with NP in RNA-dependent manner, thus delaying RNP nuclear import [46]. In contrast to TRIM25 or MOV10 RNA-dependent targeting of assembled IAV RNP, we here showed that the disruption of bunyaviral RNP by MOV10 is independent of RNA. More interestingly, MOV10 targets the small N-arm of N to block N-driven RNP assembly but does not interact with the assembled RNP, presenting a new paradigm for not only MOV10 antiviral action but also host interference with viral RNP. Although MOV10 cannot affect assemble RNP, some N oligomers, especially dimers and trimers that are uncyclized in solution, are likely able to be targeted by the host restriction factor through binding of the unoccupied N-arms as well, which would further increase the inhibitory efficiency of MOV10 and could be explored in the future. Previously, structural analyses suggested the critical role of the N-arm in N oligomerization and RNP assembly [23–25]. The present study further highlights that the N-arm and the driven N/RNP actions may represent Achilles heels of SFTSV and related bunyaviruses, which are targeted by host for antiviral defense and likewise could be exploited as targets for design of specific intervention strategies.

## Materials and methods

### Ethics statement

Animal experiments were performed in accordance to the guidelines for the Care and Use of Medical Laboratory Animals (Ministry of Health, China) and approved by the Animal Care and Use Committee as well as the Ethical Committee of Wuhan Institute of Virology, Chinese Academy of Sciences (WIVA23201801).

### Cells

HEK293T (ATCC, CRL-11268), A549 (ATCC, CCL-185), HUVEC (ATCC, CRL-1730), and Vero (ATCC, CCL-81) cells were maintained in Dulbecco’s modified Eagle’s medium (DMEM) supplemented with 10% fetal bovine serum (FBS) at 37°C under 5% CO2. HEK293 (ATCC, CRL-1573) and THP-1 (ATCC, TIB-202) cells were cultured in minimum Eagle’s medium (MEM) and Roswell Park Memorial Institute (RPMI) 1640 medium supplemented with 10% FBS, respectively. MOV10-KO or IFNAR1-KO HEK293T cells were generated by CRISPR-Cas9 gene editing as described below.

### Viruses

Severe fever with thrombocytopenia syndrome virus (SFTSV, strain WCH-2011/HN/China/isolate97), Heartland virus (HRTV, strain MO-4) and Guertu virus (GTV, strain DXM) were propagated in Vero cells for ~3–5 passages to high titers in our lab as described previously [8, 9, 14, 18, 57]. The genomic accession numbers are as follows: JQ341190.1 (SFTSV S segment), JQ341189.1 (SFTSV M segment) and JQ341188.1 (SFTSV L segment); NC_043610.1 (GTV S segment), NC_043609.1 (GTV M segment) and NC_043611.1 (GTV L segment); NC_024496.1 (HRTV S segment), NC_024494.1 (HRTV M segment), and NC_024495.1 (HRTV L segment). Bunyavirus titers were determined in Vero cells by 50% tissue culture infectious dose (TCID50) method [15, 17, 58]. Sendai virus (SeV) was propagated in 10-day-old embryonated eggs and titrated by hemagglutination assay as previously described [18].

### Plasmids and transfection

The MOV10 expression plasmid was constructed by cloning the open reading frame of MOV10 into pcDNA3.1(+) vector with an N-terminal Flag tag. Flag-tagged MOV10 mutants and S-tagged N mutants were respectively cloned into pcDNA3.1(+) and pCAGGSP7. EGFP-fused N or N-arm expression plasmids were constructed using the pEGFP-N1 vector. Expression plasmids for TBK1, SFTSV N or NSs, or HRTV N and the plasmid expressing *Renilla* luciferase (pRL-TK) were previously described [15, 16, 18, 59]. Expression plasmids for GTV N, SFTSV GP or SFTSV RdRp were similarly constructed by standard molecular biology technique. To construct the minigenome reporter plasmid transcribing the viral genome analogue (i.e. MUTR-Luc), antisense firefly luciferase gene flanked by SFTSV M-segment untranslated region (UTR) was amplified and cloned into pRF42 under the control of human polymerase I promoter. For MOV10 KD in cultured cells, the corresponding cassettes containing MOV10-targeting or control shRNA sequences (S1 Table) were cloned into the indicated pSuper.retro.puro or pLKO.1 vectors. For Mov10 KD in mice, shRNA-encoding plasmids were generated using pLV10(U6/GFP&Puro) vector. All the cloning constructs were confirmed by sequencing. Transfection of plasmids was performed with the Lipofectamine 3000 (Thermo Fisher Scientific, Cat#L3000015) or calcium phosphate (Beyotime, Cat#C0508) reagents by following the manufacturers’ instructions and recommendations. Total amount of the transfected DNA was kept constant in each group of an experiment by addition of the corresponding control plasmids.

### Antibodies

Rabbit antisera to SFTSV N, GP (GN), or RdRp were described previously [15, 17]. Mouse anti-N serum was raised against SFTSV N protein prepared from *Escherichia coli*. Mouse anti-Flag (Sigma-Aldrich, Cat#F3165), anti-HA (Sigma-Aldrich, Cat#05–904) and anti-IFNAR1 (Sigma-Aldrich, Cat#SAB1406003) antibodies and rabbit anti-MOV10 (Proteintech, Cat#10370-1-AP), anti-β-actin (ABclonal, Cat#AC026), anti-Stag (Abcam, Cat#ab18588) and anti-GFP (Abcam, Cat#ab6556) antibodies were purchased from the indicated manufacturers. For the secondary antibodies, goat anti-mouse IgG conjugated with Alexa Fluor 488 (Thermo Fisher Scientific, Cat#A-11001) and goat anti-rabbit IgG conjugated with Alexa Fluor 647 (Thermo Fisher Scientific, Cat#A32733) were used in the IFA assay; goat anti-mouse or anti-rabbit IgG antibodies conjugated with HRP (Abcam, Cat#ab6789 and Cat#ab6721) were used for IB analysis.

### Protein interaction, mass spectrometry, and IB analyses

S-pulldown assays were conducted as previously described [16, 18]. Briefly, HEK293T cells transfected with the indicated plasmids using Lipofectamine 3000 transfection reagent were lysed at 24 h posttransfection with the IB/IP lysis buffer (Beyotime, Cat#P0013). Then, the cell lysate supernatants were rotated with S-protein agarose (Millipore, Cat#69704) at 4°C for 4 h. After extensive washings of the agarose, binding proteins were eluted using 1 × sodium dodecyl sulfate (SDS) sample buffer by boiling, followed by SDS–polyacrylamide gel electrophoresis (SDS-PAGE) and mass spectrometry or IB analyses. In nuclease treatment analysis, cell lysates supplemented with 2 mM MgCl2 were divided into halves and then treated with Benzonase nuclease (100 U, Millipore, Cat#70746) or left untreated at 4°C for 2 h according to the manufacturer’s instructions before S-pulldown assays.

For the Co-IP assays with anti-N antiserum, HEK293 cells cultured in 10-cm dish were infected with SFTSV or mock infected for 24 h and lysed in the aforementioned lysis buffer. Next, the lysate supernatants were first pretreated with pre-immune serum and protein A/G agarose (Millipore, Cat#IP05) at 4°C for 1 h before centrifugation. The pretreated supernatants were then incubated with rabbit anti-N antiserum and protein A/G agarose at 4°C overnight. After extensive washings, the binding proteins on agarose were eluted by boiling as described above and analyzed by IB.

For the Co-IP assays with anti-Flag covalently conjugated to magnetic beads, cell lysates from HEK293T transfected with the indicated plasmids were incubated with anti-FLAG M2 magnetic beads (Sigma-Aldrich, Cat#M8823) at 4°C for 1 h. Following extensive washings, the samples were then eluted and analyzed by IB.

For the EGFP-nanotrap assays, HEK293T cells transfected with the indicated plasmids encoding EGFP or EGFP-fused proteins were lysed and clarified similarly. The lysate supernatants were then incubated with the EGFP-nanotrap beads (AlpaLife, Cat#KTSM1301) that are covalently coupled with nanoantibodies to EGFP at 4°C for 1 h. After extensively washing, binding proteins were eluted, followed by IB analysis.

In mass spectrometry analysis, the N or control S-pulldown products were subjected to in-gel digestion with trypsin, following SDS-PAGE [17, 60]. The trypsin-digested peptides were then analyzed by liquid chromatography-tandem mass spectrometry (LC-MS/MS) using the nano-LC-equipped TripleTOF 5600 system (AB SCIEX). Raw tandem spectra were searched against Unified Protein database (UniProt) with the ProteinPilot Software 5.0 (AB SCIEX). The obtained data were based on the false discovery rate (FDR) of ≤1% for protein identification. MOV10 peptides identified with >95% confidence and the tandem spectra of two representative peptides (>99% confidence) were shown in S1 Fig.

For IB analysis, protein samples were resolved by SDS-PAGE and transferred onto polyvinylidene difluoride (PVDF) membranes (Millipore, Cat#L3000015). After blocking with 5% bovine serum albumin (BSA) in Tris-buffered saline, the membranes were probed successively with the indicated primary antibodies and the corresponding horseradish peroxidase (HRP)-conjugated secondary antibodies. Protein bands were detected with an enhanced chemiluminescence kit (Thermo Fisher Scientific, Cat#34580) and quantitatively analyzed with the ImageJ software (National Institutes of Health) where indicated.

### Immunofluorescence and confocal microscopy

Mock- or SFTSV-infected HEK293 cells were fixed with 4% paraformaldehyde in phosphate-buffered saline (PBS) for 10 min at room temperature (RT), permeabilized with 0.5% Triton X-100 in PBS, and blocked with blocking buffer (2.5% BSA in PBS). Then, cells were incubated with the mouse anti-N (1:500) and rabbit anti-MOV10 (1:200) antibodies in blocking buffer overnight at 4°C, and stained with the fluorescein-labeled secondary antibodies for 1 h at RT. To visualize the nuclei, cells were treated with Hoechst 33258 (Beyotime, Cat#C1011) for 5 min at RT. Images were obtained using a PerkinElmer confocal microscopy (UltraVIEW VoX) and the Volocity software (PerkinElmer).

### Knockdown of MOV10 in cultured cells

For MOV10 KD in HEK293T cells, the control or MOV10-targeting shRNA plasmids constructed using the pSuper.retro.puro vector were transfected into the cells for 48 h. Transfected cells were then infected with SFTSV (0.1 MOI) for the indicated time, followed by qPCR detection of viral RNA replication in cells or growth curve analysis of viral progeny released in the supernatants.

For MOV10 KD in Vero cells, viral vectors were packaged using HEK293T cells cotransfected with the pLKO.1-based plasmids encoding control or MOV10-targeting shRNAs and the packaging plasmids (psPAX2 and pMD2.G, kindly provided by Prof. Xinwen Chen). Vero cells transduced with the packaged viral vectors were infected with SFTSV (0.1 MOI), followed by detection of KD efficiency and viral RNA replication with qPCR.

### Generation of MOV10- or IFNAR1-KO cells by CRISPR-Cas9

The guide RNA (gRNA) sequences (S1 Table) were selected with the online CRISPR Design tool (http://crispr.mit.edu/) and cloned into pX459 [61]. HEK293T cells were transfected with the constructed pX459-based plasmids for 48 h and selected with 2 μg/mL puromycin (Gibco, Cat#A1113803) for 3 days. Then, cells were diluted and seeded into 96-well plates for screening of single colony by IB and sequencing. The regions encompassing the gRNA-targeted sequences were amplified and cloned into pTOPO-Blunt vector, for sequencing analysis of the editing sites.

### Virus growth curve analysis

MOV10-KD or KO HEK293T or the control cells were incubated with SFTSV (0.1 MOI) at 4°C for 1 h. Following 3 times of washing, the cells were incubated at 37°C for the indicated time. The cell culture supernatants collected at the indicated timepoints were subjected to viral titration by TCID50 assays using Vero cells [15].

### Minigenome assay

Wildtype or IFNAR1*-*KO HEK293T cells seeded in 24-well plates were cotransfected with the SFTSV RdRp (500 ng/well) and N (250 ng/well) expression plasmids and MUTR-Luc transcription plasmid (250 ng/well), together with the plasmids expressing MOV10 (at the indicated dosages) or its truncated mutants or EGFP (500 ng/well). The total amounts of plasmids transfected in each well were kept constant by addition of control plasmids. At 48 h posttransfection, cells were subjected to measurement of firefly luciferase activity using the luciferase reporter system (Promega, Cat#E2940) on a Mithras microplate luminometer. In the dual-luciferase reporter assays for minigenome system analysis, an additional control plasmid expressing *Renilla* luciferase (pRL-TK, 5 ng/well) was also included in the transfection. Relative luciferase activities (Rel. Luc. Act.) were then shown by normalization of firefly luciferase activities to *Renilla* luciferase activities as described previously [15, 16, 59].

### Chemical cross-linking

MOV10-KO HEK293T cells were transfected with the control vector or the plasmids expressing Flag-MOV10(1–506) or Flag-MOV10(1–530), together with the N expression plasmid. At 48 h posttransfection, cells were suspended with PBS, incubated with the crosslinker disuccinimidyl suberate (DSS, 5 mM; Thermo Fisher Scientific, Cat#A39267) at RT for 15 min and quenched by 20 mM Tris, followed by SDS-PAGE resolution and IB analysis.

### RNA pulldown assay

SFTSV S-segment RNA was synthesized using T7 RNA polymerase transcription kit (Ambion, Cat#AM1314) and labeled with biotin by attaching a single biotinylated nucleotide to the 3’ terminal of the RNA strand with the Pierce RNA 3’ End Desthiobiotinylation Kit (Thermo Fisher Scientific, Cat#20163). Subsequently, 50 pmol biotion-labeled virus RNA was incubated with 50 μL streptavidin magnetic beads (Thermo Fisher Scientific, Cat#20164) at RT for 30 minutes by following the manufacturer's instructions. For pulldown assay, lysate supernatants from transfected cells were incubated with the RNA-bound magnetic beads by rotating at 4°C for 1 h, followed by washes and protein elution. The eluted samples were then subjected to SDS-PAGE and IB analysis.

### RNP extraction

RNP extraction of SFTSV was performed as previously described for that of Rift valley fever virus [62] with some modifications. HEK293 cells transfected with the Flag-MOV10 expression plasmid or control vector were infected with SFTSV (3 MOI) at 24 h posttransfection. At 24 hpi, cells were lysed in TNE buffer (Tris, 10 mM, pH 7.5; NaCl, 100 mM; EDTA, 2 mM) supplemented with 0.6% NP-40 and complete protease inhibitor (Roche, Cat#04693132001) for 30 min at 4°C. After centrifugation (1000 g; 30 min) at 4°C, the soluble supernatants were then layered on top of a discontinuous 30-40-50% CsCl (StanDard Reagent, Cat#P0481) gradient and centrifuged (38,000 rpm) in a Beckman SW41 rotor for 16 h at 4°C. The opalescent band at 40%-50% interspace was collected, followed by SDS-PAGE and IB analysis.

### Purification of SFTSV virions

SFTSV virions were concentrated and purified as previously described [3]. Culture medium of HEK293T cells infected with SFTSV was clarified by centrifugation at 3500 rpm for 10 min and then centrifuged at 28,000 rpm for 2.5 h in a Beckman SW28 rotor through 20% (wt/vol) sucrose cushion made in PBS buffer. The obtained pellet was resuspended in 500 μL PBS and applied to 15% to 45% continuous iodixanol density gradient (Sigma-Aldrich, Cat#D1556). Centrifugation of virion preparation was performed at 35,000 rpm for 2.5 h in a Beckman SW55 rotor. At the 30%-35% interface, a visible opaque band containing the virions was harvested, followed by electron microscopy and IB analyses. For electron microscopy, purified SFTSV virions were prepared using conventional negative staining protocols. Imaging was performed with 100 kV transmission electron microscope (HITACHI H-7000FA).

### iVLP packaging

HEK293T cells were cotransfected with the expression plasmids for SFTSV GP, RdRp and N, along with the MUTR-Luc transcription plasmid. At 48 h posttransfection, the cell culture medium containing the packaged iVLP was centrifugated (1,250 rpm, 5 min) and filtered with 0.45 μm filter membrane (Millipore, Cat#SLHP033RB) before usage for infection.

### Real-time qPCR

Total RNA from cultured cells or animal tissues was extracted with TRIzol (Invitrogen, Cat#15596018) for quantification of relative RNA levels normalized to the mRNA levels of human or nonhuman primate GAPDH or mouse β-actin using the 2^-ΔΔCT^ method as described previously [15, 16, 63]. The RNA in mouse serum was isolated with QIAamp RNA blood mini kit (Qiagen, Cat#52904) for absolute quantification of SFTSV S RNA by TaqMan real-time PCR using One Step PrimeScript RT-PCR kit (TaKaRa, Cat#RR064A). SFTSV S-segment RNA synthesized using T7 RNA polymerase transcription kit (Ambion, Cat#AM1314) was used as the standard to establish the standard curve. Primers for qPCR were listed in S1 Table.

### SFTSV infection of Mov10-KD adult mice

Adult female mice (6–8 weeks old) on C57/BL6 background were housed under specific-pathogen-free (SPF) conditions in the animal facility of Wuhan Institute of Virology, Chinese Academy of Sciences. Viral vectors used for KD in mice were packaged using HEK293T cells cotransfected with the shRNA-encoding pLV10(U6/GFP&Puro)-based vectors (GenePharma) and the packaging plasmids (pGag/Pol, pRev, and pVSV-G from GenePharma) by calcium phosphate method. At 48 h and 72 h posttransfection, the cell culture media were harvested and filtered with 0.45 μm filter (Millipore, Cat#SLHP033RB), followed by ultracentrifugation (20,000 rpm, 2 h, 4°C) for concentration. The pellets were resuspended with virus preservation solution and stored at -80°C for usage. Viral particle titers were measured by endpoint method [64]. For Mov10 KD *in vivo*, C57/BL6 mice (6–8 weeks old; n = 5/group) were injected with 5×10^7^ transducing units (TU) of the control or Mov10-targeting viral vectors via caudal vein [65]. Seven days posttransduction, mice were challenged with SFTSV (10^5^ TCID50) via subcutaneous injection. At 3 days postinfection, mice were sacrificed by carbon dioxide euthanasia for postmortem examination and tissue sample collection. Platelets in fresh blood treated with EDTA were counted using a hematology analyzer (Drew Scientific, Mascot HEMAVET950). Serum alanine transaminase (ALT) levels were detected by ELISA kit (Fosunpharma Diagnostics, Cat#1.02.1203). Mean values of platelet counts and ALT levels from control mice (without transduction or infection) were also detected for reference. Mov10 mRNA and viral S RNA levels in tissue samples were analyzed by qPCR as described above.

### Quantification and statistical analysis

Graphs were drawn by GraphPad Prism 8 software. All data are shown as means ± SD of n biological replicates. Statistical analyses were performed using SPSS19.0 with Student’s *t* test for the comparison between two groups and one-way analysis of variance (ANOVA) for multiple comparisons. Differences were considered statistically significant if p-value < 0.05. Significance levels are: * p < 0.05; ** p < 0.01; *** p < 0.001; **** p < 0.0001; ns, non-significant.

## Supporting information

S1 Table Oligonucleotides used in this study. (DOCX)

S1 Fig Identification of MOV10 as an N-interacting protein. HEK293 cells transfected with the control vector or the plasmid expressing S-tagged N were lysed for S-pulldown assays at 24 hours posttransfection, followed by liquid chromatography coupled with tandem mass spectrometry (LC-MS/MS) analysis of the pulldown products. MOV10 was specifically identified in the N coprecipitates but not the control pulldown products. The tandem spectra of two representative peptides (identified with > 99% confidence) of MOV10 (NP_001356436) were respectively shown. Peptides of MOV10 identified with high confidence (> 95%) were summarized in the bottom table.
(TIF)

S2 Fig RNA-independent interactions of MOV10 with the N proteins of SFTSV-related bunyaviruses. HEK293T cells transfected with the plasmids encoding Flag-MOV10 and the indicacted S-tagged N proteins of GTV (GN-Stag) or HRTV (HN-Stag) were lysed at 24 h posttransfection and treated with Benzonase or left untreated, followed by S-pulldown and IB analyses as in Fig 1F.
(TIF)

S3 Fig Induction of MOV10 and typical ISGs in HEK293 cells. (A-H) IFN-α cannot noticeably stimulate MOV10 expression in HEK293 cells. HEK293 cells were treated with the indicated concentrations of IFN-α for 6h (A-D) or with 100 U/mL IFN-α for the indicated time (E-H). The mRNA levels of MOV10 and several classical ISGs, ISG15, myxovirus-resistance A (MxA), and oligoadenylate synthetase 1 (OAS1), were then analyzed by real-time qPCR. (I-K) SeV-stimulated expression of the typical ISGs. HEK293 cells were infected with SeV or mock infected for the indicated time, followed by analyses of the indicated ISG mRNA levels by qPCR. Data were shown as mean ± SD, n = 3. ns, non-significant. Related to Fig 2.
(TIF)

S4 Fig Induction of MOV10 and other ISGs in THP-1, HUVEC, and A549 cells. (A-H) IFN-α can only trigger weak and transient induction of MOV10 in THP-1 monocytes. THP-1 cells were treated with the indicated concentrations of IFN-α for 6h (A-D) or with 100 U/mL IFN-α for the indicated time (E-H), followed by analyses of MOV10 and the classical ISG mRNA levels with qPCR. (I-P) IFN-α-induced MOV10 expression in HUVEC and A549 cells. Similarly, HUVEC (I-L) or A549 (M-P) cells were treated with the indicated concentrations of IFN-α (I, J, M, and N) or with 100 U/mL IFN-α for the indicated time (K, L, O, and P), followed by analyses of the indicated ISG mRNA levels by qPCR. Data were shown as mean ± SD, n = 3. Related to Fig 2.
(TIF)

S5 Fig Validation of MOV10-KO or IFNAR1-KO HEK293T cells generated by CRISPR-Cas9. DNA sequencing and IB analyses of *MOV10*-KO (A and B) or *IFNAR1*-KO (C and D) HEK293T cells. Regions encompassing the second exon of MOV10 or the first exon of IFNAR1 were respectively amplified by PCR. The PCR products were TA-cloned into pTOPO-Blunt (Aidlab #CV16), followed by sequencing of at least seven individual clones using the M13F sequencing primer. The PAM sequences are indicated in dashed boxes and resulting protein sequences are shown below the aligned DNA sequences (A and C). To validate the results at protein expression levels, the generated cells were subjected to IB analyses with the indicated antibodies (B and D). See also the Materials and Methods for experimental details.
(TIF)

S6 Fig SFTSV infection in non-transduced or the control viral vector-transduced mice. C57/BL6 mice were injected with the viral vector encoding control shRNA (5×10^7^ TU, n = 4) or the storage solution (n = 3) via caudal vein, followed by infection with SFTSV, 7 d posttransduction, as in Fig 4. Three days postinfection, mice were sacrificed for evaluation of viral S RNA copies in the indicated organs (A, spleen; B, liver; C, lung). Data are means ± SD. ns, non-significant. Related to Fig 4.
(TIF)

S7 Fig RNP reconstitution minigenome assays with dual-luciferase reporter system. HEK293T (A and C) or the IFN receptor-KO HEK293T cells (B) were respectively used to assess suppression of SFTSV RNP by MOV10 or the truncated proteins with the RNP reconstitution minigenome system similarly to Figs 5A, 5B, or 6E, with the exception that an additional control plasmid (pRL-TK) was also included in these transfection assays. At 48 h posttransfection, cells were delivered to dual luciferase activity measurement. Relative luciferase activities (Rel. Luc. Act.) are shown. Data are means ± SD, n = 3.
(TIF)

S8 Fig MOV10(1–100) does not noticeably inhibit N polymerization. MOV10-KO HEK293T cells were transfected with the Flag-tagged MOV10(1–100) expression plasmid or control vector, together with the SFTSV N expression plasmid, and cross-linked with DSS as in Fig 7E and 7F, followed by SDS-PAGE and IB analyses. The band intensities of polymers and monomers were quantified using ImageJ and then the relative intensities over the controls were respectively calculated.
(TIF)

S9 Fig RNA pulldown assays without N expression. RNA pulldown assays were performed similarly as in Fig 7G, with the exception that the N expression plasmid was replaced by control vector in the transfection. Cell lysate inputs and pulldown products were then analyzed by IB.
(TIF)

S10 Fig MOV10 does not bind to SFTSV virions harboring assembled RNPs or affect the action of assembled RNPs in incoming iVLP. (A) Experimental procedure for SFTSV virion purification. The culture supernatant of HEK293 cells infected with SFTSV was clarified by centrifugation and subjected to ultracentrifugation as described in Materials and Methods. The fraction containing virions were harvested and used for the following analyses. (B) Electron micrograph showing purified SFTSV virions. (C) Purified virions and lysates of infected cells were subjected to IB analyses with the indicated antibodies. (D) MOV10 does not affect the functioning of assembled RNPs in incoming iVLP. HEK293T were cotransfected with the plasmids expressing SFTSV RdRp, N, and GP, along with the MUTR-Luc transcription plasmid. The cell culture supernatant containing iVLP was harvested at 48 h posttransfection and used to infect the HEK293 cells which was transfected with the plasmids expressing full-length MOV10(1–1003) or truncated MOV10(1–506), 24 h prior to the iVLP infection. The iVLP-infected cells were then delivered to luciferase activity measurement and protein expression detection by IB. Data are means ± SD, n = 3. ns, non-significant.
(TIF)
