# Supplementary figures and images for: Host restriction of emerging high-pathogenic bunyaviruses via MOV10 by targeting viral nucleoprotein and blocking ribonucleoprotein assembly
Source: PLoS Pathog. 2020 Dec 7;16(12):e1009129. doi: 10.1371/journal.ppat.1009129 (PMC7746268; doi:10.1371/journal.ppat.1009129)

**Oligonucleotides used in this study.**


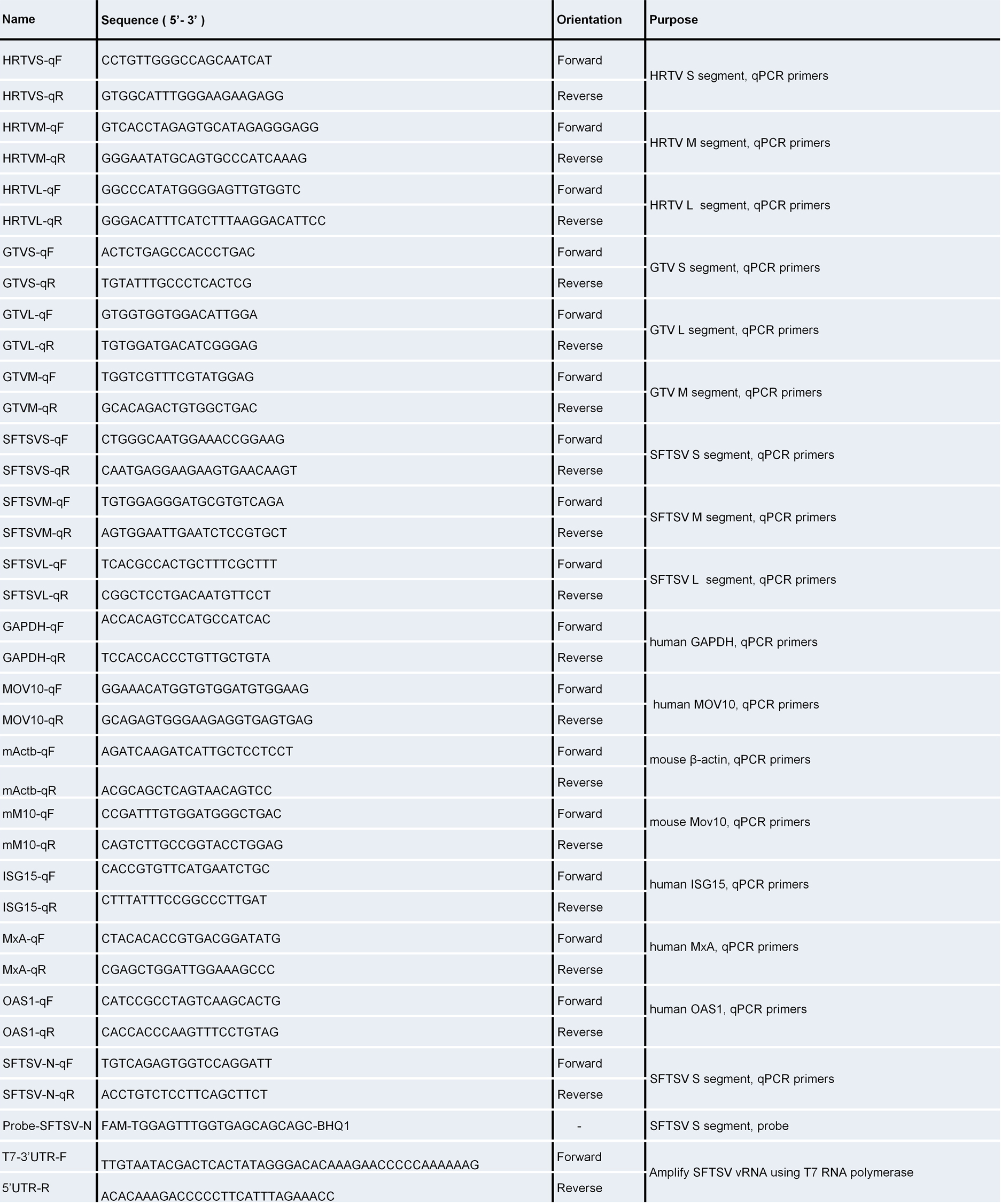

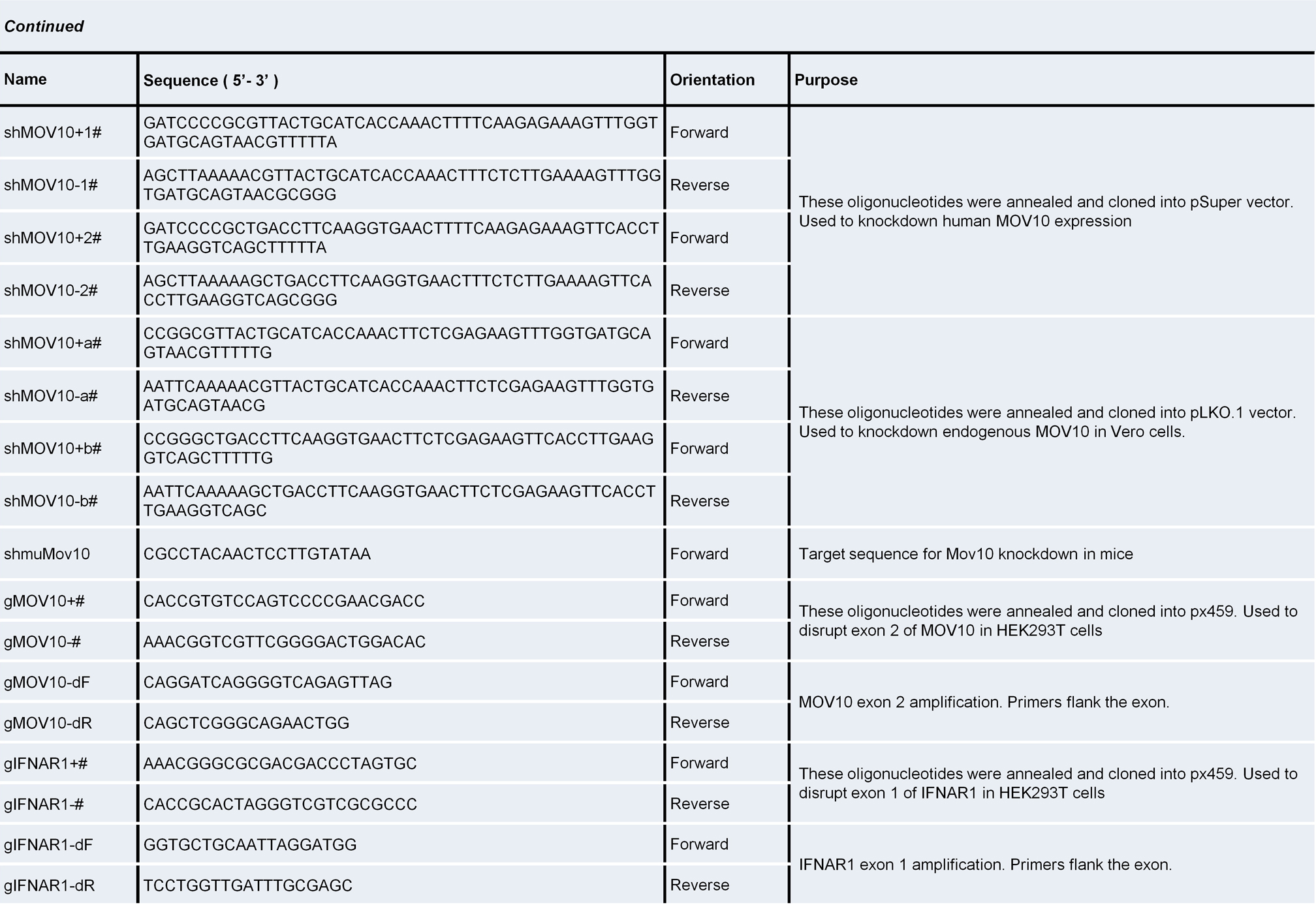

Supplement: S1 Table — (DOCX) [file ppat.1009129.s001.docx]

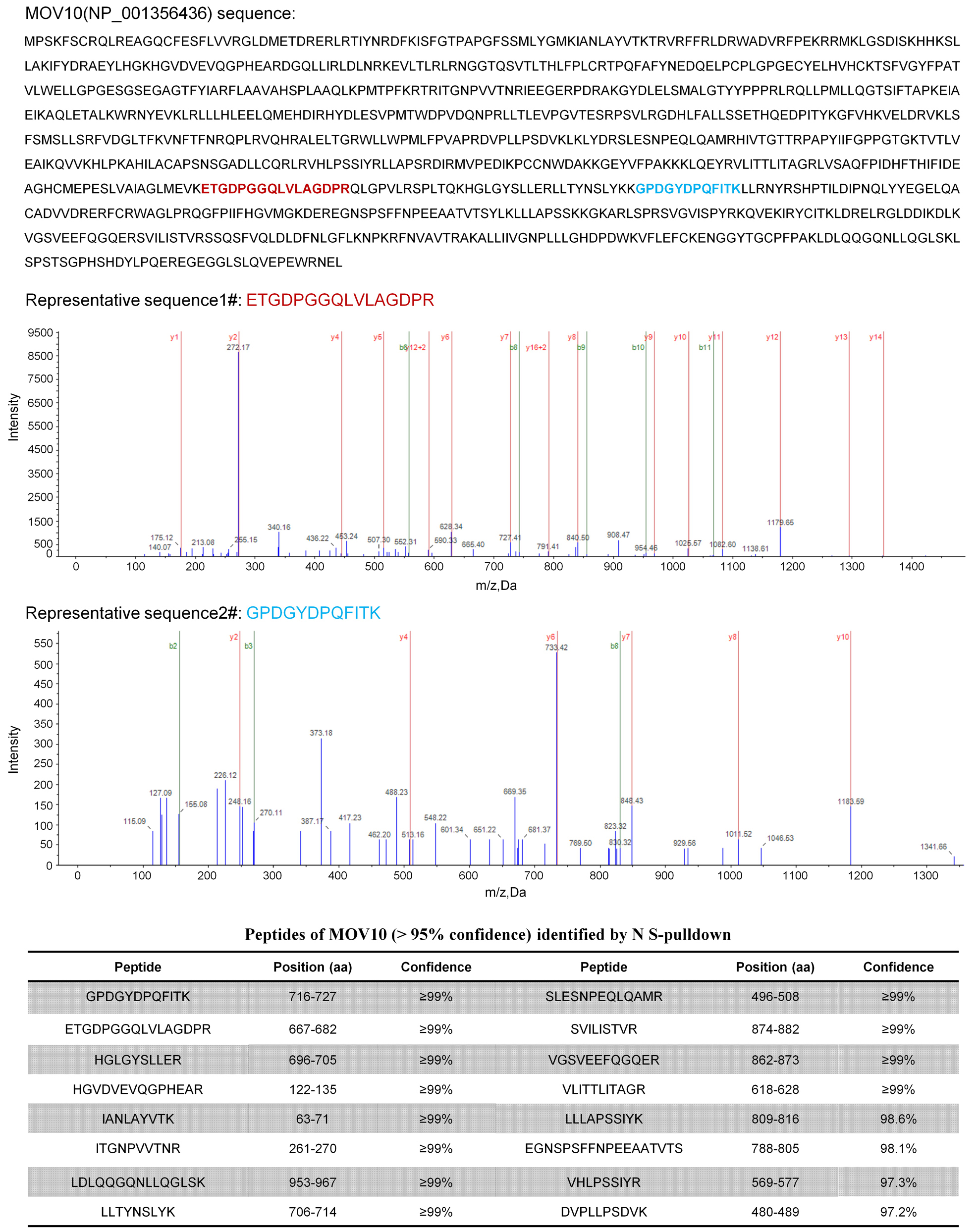

Supplement: S1 Fig — HEK293 cells transfected with the control vector or the plasmid expressing S-tagged N were lysed for S-pulldown assays at 24 hours posttransfection, followed by liquid chromatography coupled with tandem mass spectrometry (LC-MS/MS) analysis of the pulldown products. MOV10 was specifically identified in the N coprecipitates but not the control pulldown products. The tandem spectra of two representative peptides (identified with > 99% confidence) of MOV10 (NP_001356436) were respectively shown. Peptides of MOV10 identified with high confidence (> 95%) were summarized in the bottom table. (TIF) [file ppat.1009129.s002.tif]

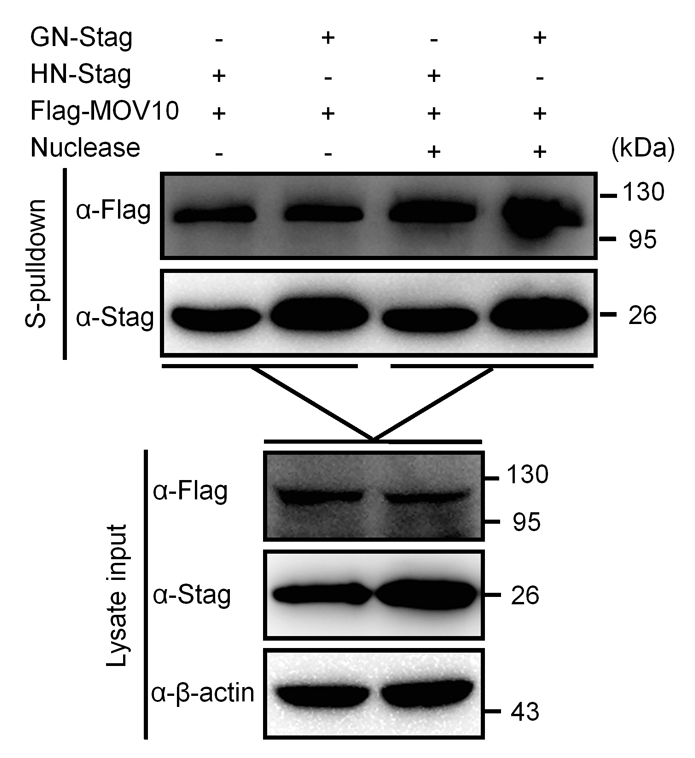

Supplement: S2 Fig — HEK293T cells transfected with the plasmids encoding Flag-MOV10 and the indicacted S-tagged N proteins of GTV (GN-Stag) or HRTV (HN-Stag) were lysed at 24 h posttransfection and treated with Benzonase or left untreated, followed by S-pulldown and IB analyses as in Fig 1F. (TIF) [file ppat.1009129.s003.tif]

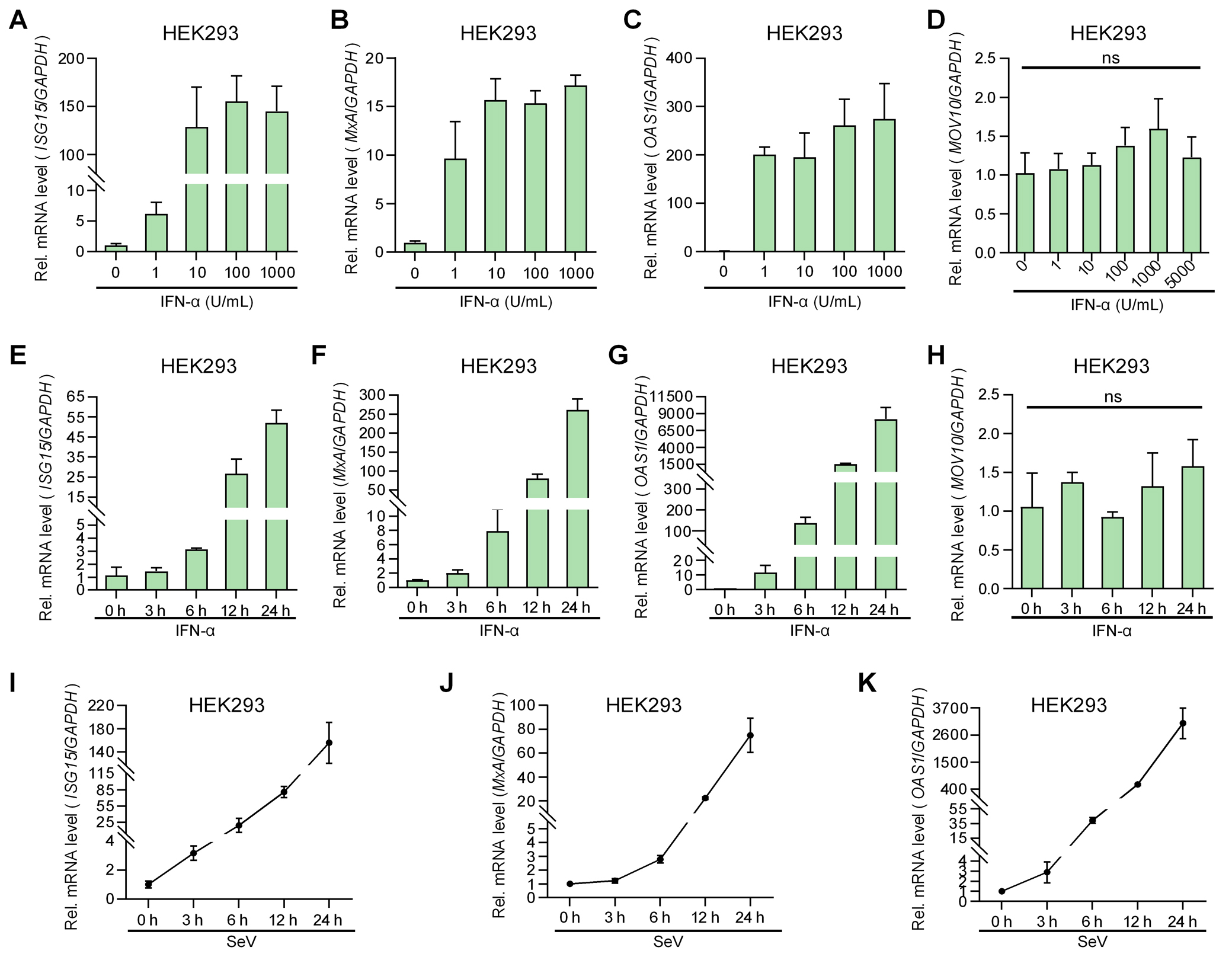

Supplement: S3 Fig — (A-H) IFN-α cannot noticeably stimulate MOV10 expression in HEK293 cells. HEK293 cells were treated with the indicated concentrations of IFN-α for 6h (A-D) or with 100 U/mL IFN-α for the indicated time (E-H). The mRNA levels of MOV10 and several classical ISGs, ISG15, myxovirus-resistance A (MxA), and oligoadenylate synthetase 1 (OAS1), were then analyzed by real-time qPCR. (I-K) SeV-stimulated expression of the typical ISGs. HEK293 cells were infected with SeV or mock infected for the indicated time, followed by analyses of the indicated ISG mRNA levels by qPCR. Data were shown as mean ± SD, n = 3. ns, non-significant. Related to Fig 2. (TIF) [file ppat.1009129.s004.tif]

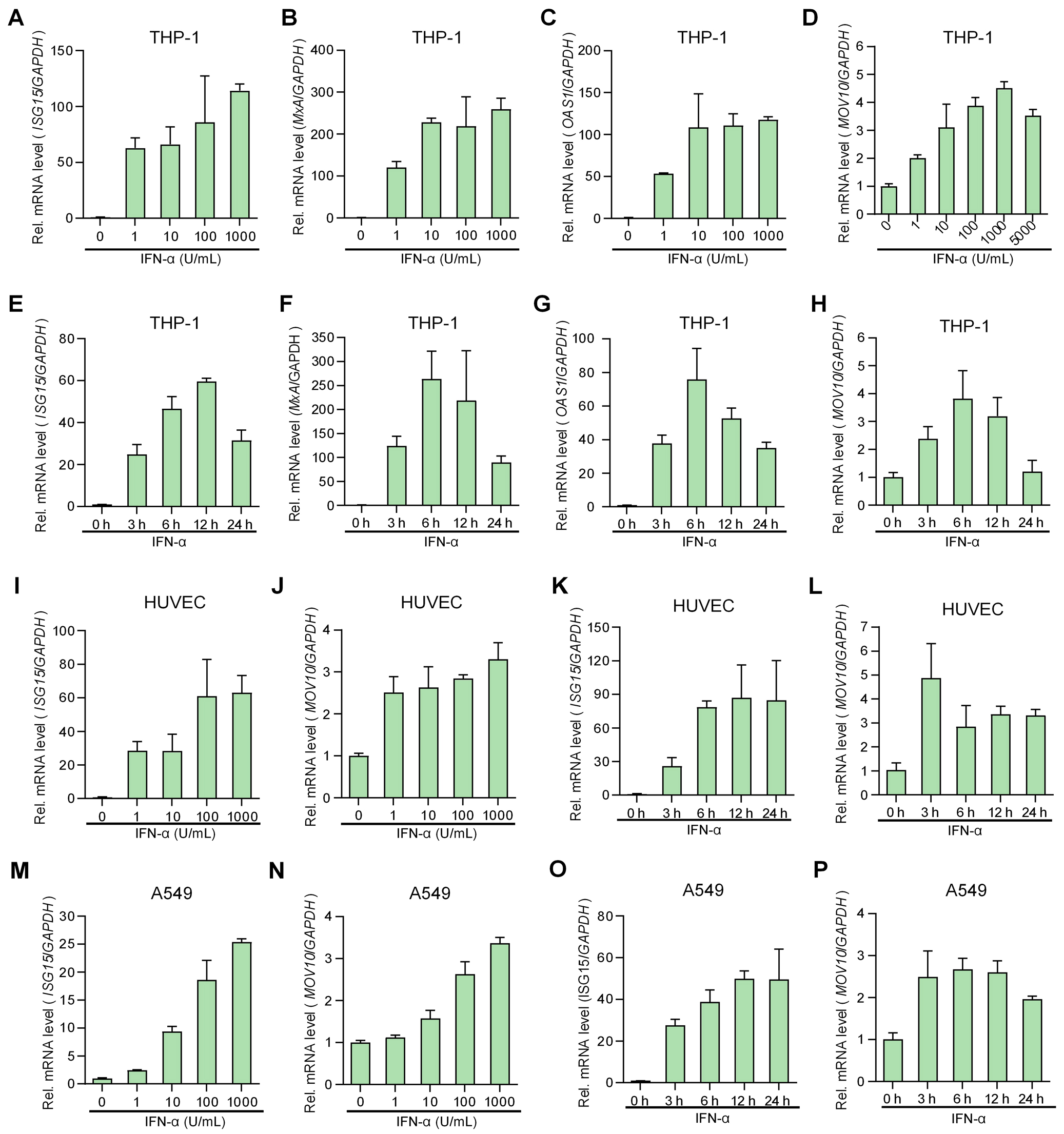

Supplement: S4 Fig — (A-H) IFN-α can only trigger weak and transient induction of MOV10 in THP-1 monocytes. THP-1 cells were treated with the indicated concentrations of IFN-α for 6h (A-D) or with 100 U/mL IFN-α for the indicated time (E-H), followed by analyses of MOV10 and the classical ISG mRNA levels with qPCR. (I-P) IFN-α-induced MOV10 expression in HUVEC and A549 cells. Similarly, HUVEC (I-L) or A549 (M-P) cells were treated with the indicated concentrations of IFN-α (I, J, M, and N) or with 100 U/mL IFN-α for the indicated time (K, L, O, and P), followed by analyses of the indicated ISG mRNA levels by qPCR. Data were shown as mean ± SD, n = 3. Related to Fig 2. (TIF) [file ppat.1009129.s005.tif]

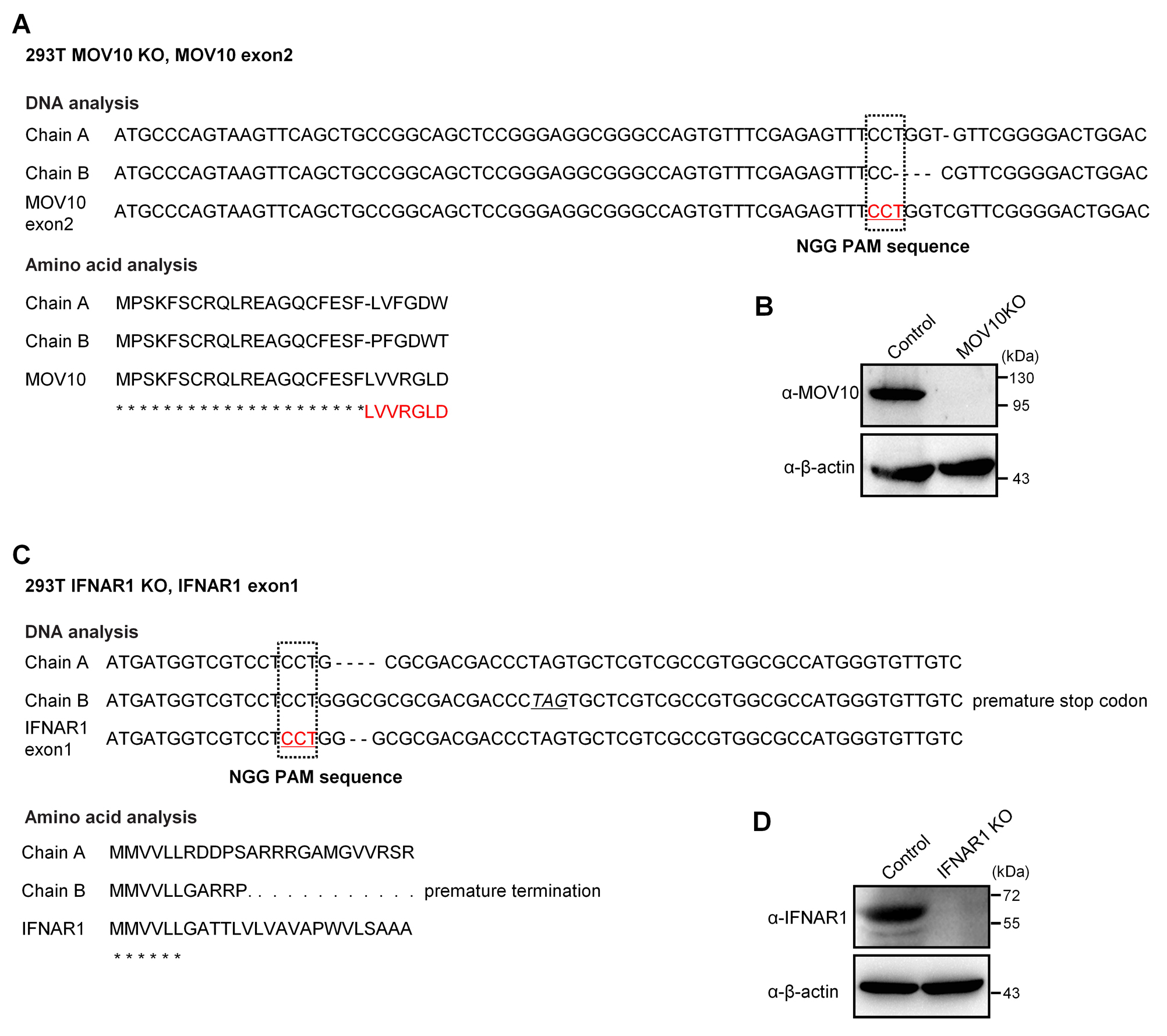

Supplement: S5 Fig — DNA sequencing and IB analyses of MOV10-KO (A and B) or IFNAR1-KO (C and D) HEK293T cells. Regions encompassing the second exon of MOV10 or the first exon of IFNAR1 were respectively amplified by PCR. The PCR products were TA-cloned into pTOPO-Blunt (Aidlab #CV16), followed by sequencing of at least seven individual clones using the M13F sequencing primer. The PAM sequences are indicated in dashed boxes and resulting protein sequences are shown below the aligned DNA sequences (A and C). To validate the results at protein expression levels, the generated cells were subjected to IB analyses with the indicated antibodies (B and D). See also the Materials and Methods for experimental details. (TIF) [file ppat.1009129.s006.tif]

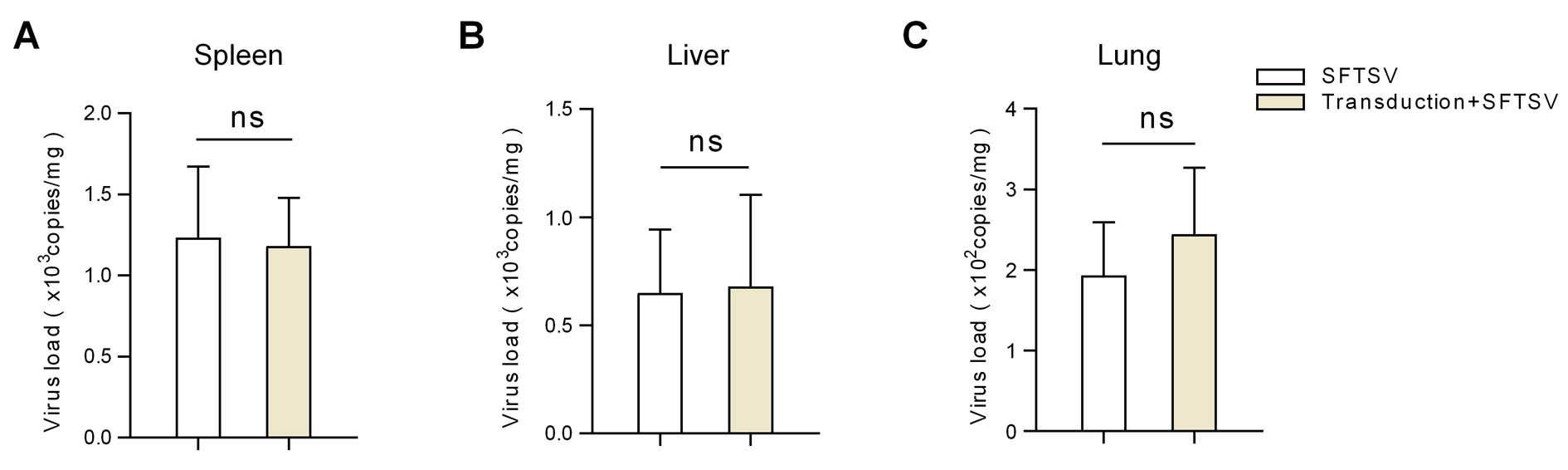

Supplement: S6 Fig — C57/BL6 mice were injected with the viral vector encoding control shRNA (5×10^7^ TU, n = 4) or the storage solution (n = 3) via caudal vein, followed by infection with SFTSV, 7 d posttransduction, as in Fig 4. Three days postinfection, mice were sacrificed for evaluation of viral S RNA copies in the indicated organs (A, spleen; B, liver; C, lung). Data are means ± SD. ns, non-significant. Related to Fig 4. (TIF) [file ppat.1009129.s007.tif]

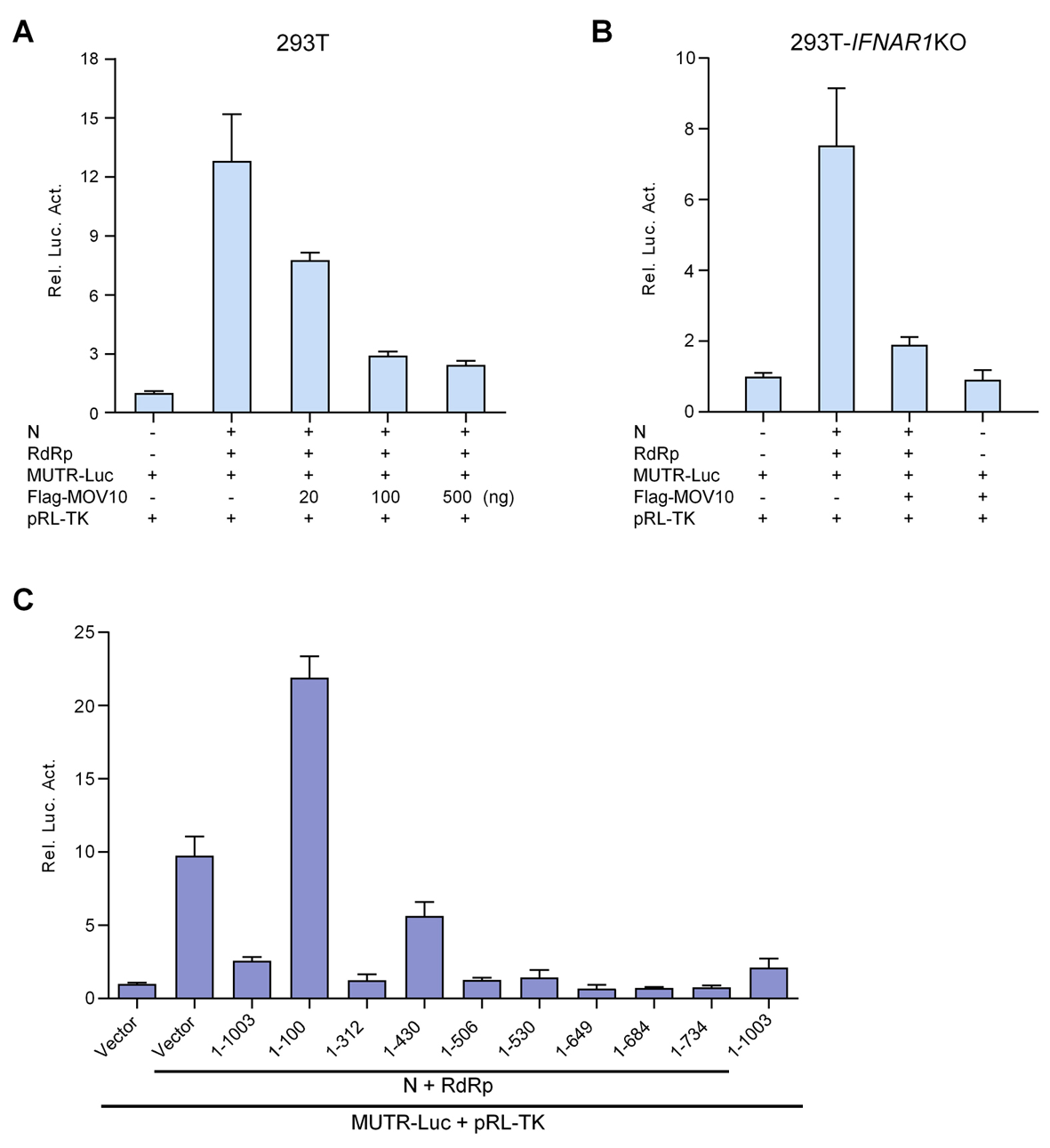

Supplement: S7 Fig — HEK293T (A and C) or the IFN receptor-KO HEK293T cells (B) were respectively used to assess suppression of SFTSV RNP by MOV10 or the truncated proteins with the RNP reconstitution minigenome system similarly to Figs 5A, 5B, or 6E, with the exception that an additional control plasmid (pRL-TK) was also included in these transfection assays. At 48 h posttransfection, cells were delivered to dual luciferase activity measurement. Relative luciferase activities (Rel. Luc. Act.) are shown. Data are means ± SD, n = 3. (TIF) [file ppat.1009129.s008.tif]

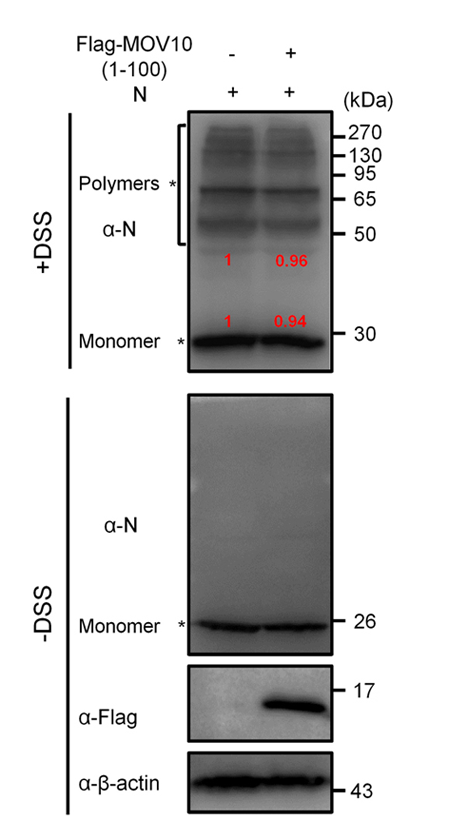

Supplement: S8 Fig — MOV10-KO HEK293T cells were transfected with the Flag-tagged MOV10(1–100) expression plasmid or control vector, together with the SFTSV N expression plasmid, and cross-linked with DSS as in Fig 7E and 7F, followed by SDS-PAGE and IB analyses. The band intensities of polymers and monomers were quantified using ImageJ and then the relative intensities over the controls were respectively calculated. (TIF) [file ppat.1009129.s009.tif]

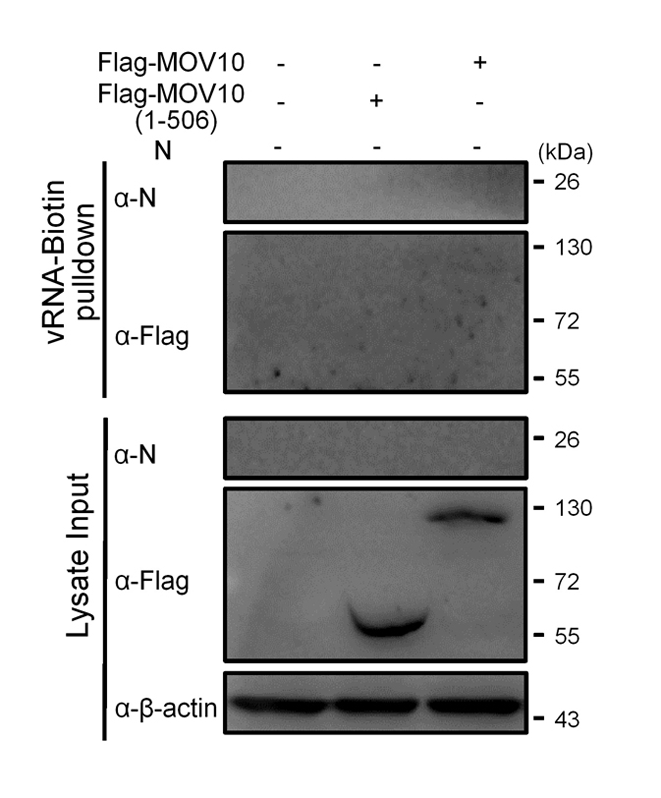

Supplement: S9 Fig — RNA pulldown assays were performed similarly as in Fig 7G, with the exception that the N expression plasmid was replaced by control vector in the transfection. Cell lysate inputs and pulldown products were then analyzed by IB. (TIF) [file ppat.1009129.s010.tif]

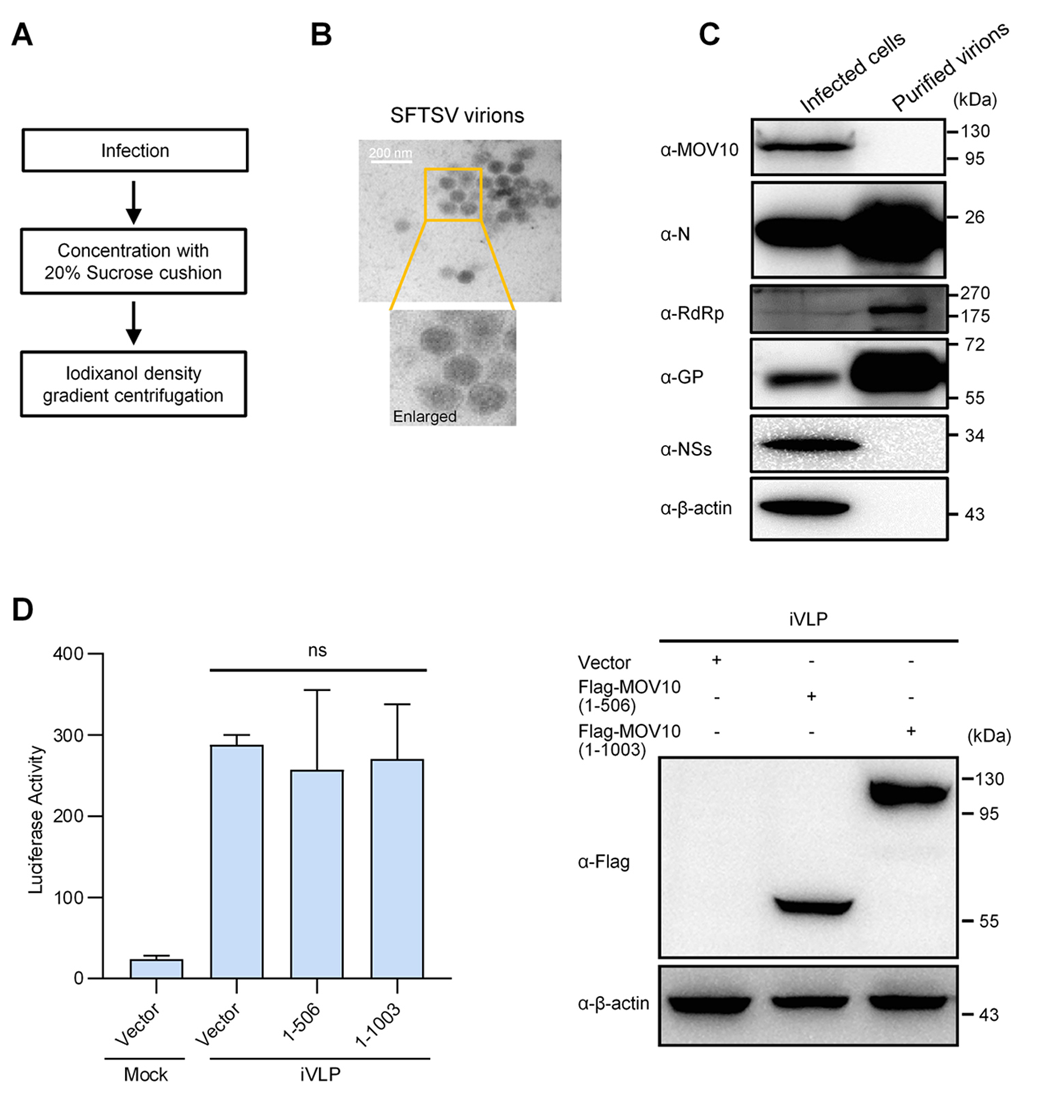

Supplement: S10 Fig — (A) Experimental procedure for SFTSV virion purification. The culture supernatant of HEK293 cells infected with SFTSV was clarified by centrifugation and subjected to ultracentrifugation as described in Materials and Methods. The fraction containing virions were harvested and used for the following analyses. (B) Electron micrograph showing purified SFTSV virions. (C) Purified virions and lysates of infected cells were subjected to IB analyses with the indicated antibodies. (D) MOV10 does not affect the functioning of assembled RNPs in incoming iVLP. HEK293T were cotransfected with the plasmids expressing SFTSV RdRp, N, and GP, along with the MUTR-Luc transcription plasmid. The cell culture supernatant containing iVLP was harvested at 48 h posttransfection and used to infect the HEK293 cells which was transfected with the plasmids expressing full-length MOV10(1–1003) or truncated MOV10(1–506), 24 h prior to the iVLP infection. The iVLP-infected cells were then delivered to luciferase activity measurement and protein expression detection by IB. Data are means ± SD, n = 3. ns, non-significant. (TIF) [file ppat.1009129.s011.tif]
